# Time-resolved transcriptomic analysis reveals envelope stress–mediated adaptation of *Escherichia coli* to the LPS transport inhibitor thanatin

**DOI:** 10.3389/fmicb.2026.1920608

**Published:** 2026-09-30

**Authors:** Renu Bisht, Elisabete C. C. M. Moura, Julien Prados, Alessandra Romanelli, Alessandra M. Martorana, Pierre D. Charlesworth, Mélanie Roch, William L. Kelley, Paola Sperandeo, Alessandra Polissi

**Affiliations:** 1 Department of Pharmacological and Biomolecular Sciences “Rodolfo Paoletti”, University of Milano, Milano, Italy; 2 Bioinformatics Support Platform, Faculty of Medicine, University of Geneva, Geneva, Switzerland; 3 Department of Pharmaceutical Sciences, University of Milano, Milano, Italy; 4 Department of Microbiology and Molecular Medicine, University Hospital and Medical School of Geneva, Geneva, Switzerland

**Keywords:** thanatin, antimicrobial peptides, lipopolysaccharide transport, envelope stress response, Cpx signal transduction, RNA-seq, antimicrobial resistance

## Abstract

Gram-negative bacteria are a major cause of antibiotic-resistant infections due to their highly impermeable cell envelope which contains lipopolysaccharide (LPS) and prevents antibiotics from entering the cell. LPS biogenesis relies on the activity of the essential LPS transport machinery (Lpt), which represents an attractive and validated target for antibiotic development. The antimicrobial peptide thanatin impairs LPS transport by disrupting the assembly of the Lpt protein machinery, resulting in cell death. However, thanatin is not a suitable drug candidate due to its poor drug-like properties and the rapid emergence of resistance. Therefore, understanding the mechanisms underlying cell resistance and adaptation to thanatin is crucial for developing novel antimicrobial strategies. Here we performed RNA-seq–based kinetic transcriptomic analysis to investigate how bacterial cells respond to thanatin over time followed by a functional validation. We found that thanatin treatment induces a rapid activation of the Cpx envelope stress response system, followed by activation of Rcs phosphorelay and downregulation of the FlhDC regulon. Cpx regulated genes showed enrichment for pathways involved in cationic peptide resistance, stress regulation, protein folding, and peptidoglycan remodelling. Rcs upregulated genes were associated with capsule synthesis and biofilm formation. Finally, late response to thanatin suggests the involvement of phospholipid metabolism modulation and trafficking as a strategy to restore OM asymmetry. Overall, our results delineate the adaptive mechanisms employed by bacterial cells to counteract thanatin-induced stress through an interplay of multiple regulatory pathways controlling envelope integrity, stress responses, motility and biofilm formation.

## 1 Introduction

Despite the transformative impact of antibiotics on medicine over the last century, infectious diseases are once again emerging as a major threat to global health. This reappearance is primarily driven by the emergence and spread of drug-resistant bacterial strains, largely resulting from antibiotic misuse (Murray et al., 2022). In recognition of this growing crisis, the World Health Organization (WHO) identified infections caused by multi-drug resistant (MDR) bacterial pathogens as a global health priority in 2017, with projections estimating that up to 10 million deaths annually would be attributable to MDR infections by 2050 (de Kraker et al., 2016). Despite these alarming predictions, antibiotic discovery and development have progressed slowly, underscoring the urgent need for alternative strategies to combat MDR pathogens. The scenario is even more challenging for infections caused by Gram-negative pathogens, which resist entry by many antibiotics commonly effective against Gram-positive bacteria due to a characteristic cell envelope architecture (Nikaido, 2003; Silhavy et al., 2010). This envelope architecture comprises the cytoplasmic inner membrane (IM), an aqueous periplasmic space containing the peptidoglycan cell wall (PG), and, external to both, an asymmetric outer membrane (OM) (Silhavy et al., 2010). The OM consists of an inner layer of phospholipids and an outer layer of tightly packed lipopolysaccharide (LPS) molecules. The amphipathic nature of LPS restricts the permeation of many hydrophobic and large molecules through the OM (Nikaido, 2003).

Overcoming this permeability barrier calls for new antimicrobial treatment options, which may arise from the mining of previously untapped sources of bioactive molecules -including antimicrobial peptides (AMPs)- as well as from the exploitation of new bacterial targets. AMPs are a class of antimicrobial molecules that act as a first line of defence against a wide range of bacteria, fungi, and viruses through diverse mechanisms of action (Hancock and Sahl, 2006). The cationic nature of AMPs enables their interaction with negatively charged bacterial membranes, leading to membrane destabilization through pore formation or by promoting infection clearance via the activation of the host adaptive immune system—properties that make AMPs promising antimicrobial agents (Zasloff, 2002). Accordingly, most AMPs exert their bactericidal effects by disrupting bacterial membranes, a strategy that may also hamper the development of antimicrobial resistance in microorganisms (Erdem Buyukkiraz and Kesmen, 2022); among these membrane-active peptides is thanatin.

Thanatin is a 21-residue AMP, derived from the hemipteran insect *Podisus maculiventris*, that belongs to the membrane agglutinating AMP category (Dash and Bhattacharjya, 2021; Fehlbaum et al., 1996; Vetterli et al., 2018). Thanatin exhibits broad spectrum antifungal and antibacterial activities, with higher potency against Gram-negative bacteria, particularly members of the *Enterobacteriaceae* family, including drug-resistant strains (Ma et al., 2019). Detailed analyses of the physical and biological properties of thanatin have revealed a dual mechanism of action. According to the proposed model, following its initial interaction with the bacterial surface, thanatin inserts into and permeates through the OM, reaching the periplasm where it binds its molecular target. LPS acts as the negatively charged receptor for thanatin on the bacterial cell surface (Sinha et al., 2022). Within the periplasm, thanatin targets the LPS transport (Lpt) multiprotein complex (Moura et al., 2020; Vetterli et al., 2018). In *Escherichia coli*, the Lpt complex consists of seven essential core proteins (LptA-G) and two accessory proteins (LptM and YedD) that form a trans-envelope bridge connecting the IM and the OM, thereby mediating the ATP-dependent transport of newly synthesized LPS across the periplasm and assembling it at the OM (Gennaris et al., 2025; Lundstedt et al., 2021; Sperandeo et al., 2019; Okuda et al., 2016; Yang et al., 2023). Given the essential role of the Lpt machinery in bacterial survival, AMPs targeting this system, such as thanatin, represent promising antimicrobial candidates.

Structural and genetic evidence supports this candidacy. Thanatin binds to the N-terminal ß-strand of the periplasmic LptA component and predominantly disrupts the interaction between LptA and LptC (Vetterli et al., 2018; Moura et al., 2020). It has also been shown to interact with the OM protein LptD, which, in complex with LptE, constitutes the OM translocon responsible for the final step of LPS assembly at the cell surface (Freinkman et al., 2011). This interaction stabilizes a specific conformation of LptD *in vitro,* thereby preventing LPS release from the translocon (Fiorentino et al., 2021). Initial insights into the molecular target of thanatin were obtained through the analysis of suppressor mutants isolated following incubation of *E. coli* cells with the peptide. These studies identified a point mutation in *lptA,* suggesting that LptA is the primary target of thanatin activity (Vetterli et al., 2018). However, the ease with which suppressor mutations could be isolated suggests that, despite its potential, thanatin is not an ideal antibiotic candidate and requires further optimization.

Since then, several analogues have been synthesized to broaden the spectrum of activity against difficult-to-treat pathogens (such as *Acinetobacter baumannii*; Oi et al., 2023) or to reduce the emergence of resistance (Schuster et al., 2023); however, none have yet reached the clinic, suggesting that a more in-depth understanding of the mechanism underlying bacterial resistance to thanatin is needed. One plausible route is activation of envelope stress response systems: exposure to antimicrobial peptides is a known inducer of these systems (Hews et al., 2019), and, more broadly, bacteria develop resistance to antibiotics by activating stress response systems that stimulate adaptive mechanisms, repair cellular damage, promote resistance lifestyles, or induce the emergence of resistance mutations (Poole, 2012). Consequently, an alternative to medicinal chemistry approaches for improving antibiotic efficacy is to interfere with bacterial adaptation and resistance mechanisms.

In Gram-negative bacteria, the integrity of the cell envelope is monitored by dedicated stress response systems (Mitchell and Silhavy, 2019). Envelope stress response systems (ESRs) detect envelope damage and respond by altering the transcriptome to restore and maintain envelope integrity (Bisht et al., 2024). The most relevant ESRs are Cpx, Rcs and σ^E^, which sense and respond to partially overlapping triggers arising from chemical or environmental challenge. Though less consequential compared with the aforementioned ESRs, other stress response systems also monitor cell envelope integrity, including the two-component systems PhoPQ and PmrAB (also named BasSR in *E. coli*), that control the expression of LPS-modifying enzymes (Chen and Groisman, 2013; Prost and Miller, 2008).

The Cpx (conjugative pilus expression) response plays a primary role in protecting IM integrity (Mitchell and Silhavy, 2019). The Cpx system is activated by conditions that cause defects in protein secretion across the IM, impaired PG synthesis, and the accumulation of misfolded proteins in the IM and periplasm (Mitchell and Silhavy, 2019). AMPs have been shown to induce this system (Hews et al., 2019).

The Rcs (regulator of capsule synthesis) system is sensitive to changes in LPS net charge or LPS layer fluidity, responds to defects in lipoprotein trafficking, PG biosynthesis, and senses the loss of periplasmic osmoprotectant sugars (Mitchell and Silhavy, 2019). These cues induce the expression of genes involved in the production of colanic acid (CA) capsule and biofilm formation, while genes involved in motility and flagella production are repressed (Ferrieres et al., 2007; Francez-Charlot et al., 2003; Wehland and Bernhard, 2000). Specifically, overproduction of the anionic CA reinforces protection against cationic AMPs that disrupt the LPS surface layer (Gruenheid and Le Moual, 2012).

Finally, the σ^E^ stress response system is sensitive to perturbations of the OM, such as periplasmic accumulation of misfolded OM proteins (OMPs) or mislocalized and modified LPS (Danese and Silhavy, 1997; Klein et al., 2009; Lima et al., 2013; Tam and Missiakas, 2005). Other, more general stimuli—including oxidative stress, heat shock, acid stress, biofilm formation, hypo-osmotic shock, and carbon starvation—have also been shown to induce this stress response system (Hews et al., 2019). Induction of this stress response ultimately activates the transcription of genes involved in the biosynthesis, transport and/or assembly of OM components (OMPs, LPS, phospholipids), as well as periplasmic chaperones and proteases (Combs and Silhavy, 2024; Mitchell and Silhavy, 2019).

In this work we investigated the global transcriptional changes in thanatin-treated *E. coli* cells at an early point and after one generation. Analysis of the stress response systems activated upon membrane perturbation and disruption of Lpt assembly by thanatin, together with the identification of differentially regulated genes, reveals new functions that can be targeted in a combinatorial approach to increase the potency of this promising AMP.

## 2 Materials and methods

### 2.1 Bacterial strains and growth conditions

All *E. coli* strains used in this study are listed in Table 1. Knockout mutants were generated by P1 transduction transferring the desired mutant alleles from the corresponding *kan*-marked alleles from the Keio *E. coli* single-gene knockout library (Baba et al., 2006) into wild-type MG1655 as previously described (Silhavy et al., 1984). Resistance cassette insertion was verified by PCR. The BW25113 strain was used for β-galactosidase assay and CA determination, while MG1655 was used for MIC determination, RNA sequencing, and physiological studies.

**Table 1:** Strains and plasmids.

Strain/plasmid	Description	Source or reference
Strains		
MG1655	K-12, F^−^ λ^−^ *ilvG*^−^ *rfb-50 rph-1*	Blattner et al., 1997
BW25113	*lacI^q^ rrnB_T14_ ΔlacZ_WJ16_ hsdR514* Δ*araBAD_AH33_* Δ*rhaBAD_LD78_*	Datsenko and Wanner, 2000
XL1-Blue	*recA1 endA1 gyrA96 (Nal^R^) thi-1 hsdR17 supE44 relA1 lac* [*F′ proAB lacI^q^*Z ∆*M15* Tn*10* (Tet^R^)]	
BW25113∆*wcaJ*	Δ*wcaJ::cat*	Laboratory collection
JW2669	∆*recA::kan*	Baba et al., 2006
JW2205	∆*rcsB::kan*	Baba et al., 2006
JW3883	∆*cpxR::kan*	Baba et al., 2006
MG1655∆*cpxR*	Δ*cpxR::kan*	This study
MG1655∆*rcsB*	Δ*rcsB::kan*	This study
MG1655∆*recA*	*ΔrecA::kan*	This study
Plasmids		
pRS415	pBR322 derivative; harbors the entire *lac* operon without promoter; Amp^R^	Simons et al., 1987
pRS415-*cpxPp*	pRS415 derivative, expresses LacZ from the *cpxP* promoter region; Amp^R^	This study
pRS415-*rprAp*	pRS415 derivative, expresses LacZ from the *rprA* promoter region; Amp^R^	This study
pRS415-*rpoEp*	pRS415 derivative, expresses LacZ from the *rpoE* promoter region; Amp^R^	This study

Bacteria were grown aerobically at 37 °C on LB plates or in liquid LB medium (10 g/L of tryptone, 5 g/L of yeast extract, 10 g/L of NaCl; 10 g/L of agar for plates). Antibiotics were used at the following concentrations: chloramphenicol (Cam), 25 μg/mL; kanamycin (Kan), 25 μg/mL; ampicillin (Ap), 100 μg/mL.

Thanatin was synthesized on a 0.1 mmol scale as previously described (Moura et al., 2020).

The minimal inhibitory concentration (MIC) of thanatin was determined using the broth dilution method described in (Wiegand et al., 2008). Multiple batches of thanatin were synthesized during the study, with MIC values determined for each batch ranging from 1.8 to 3.5 μg/mL.

### 2.2 Plasmid construction

Plasmids and oligonucleotides used in this study are shown in Tables 1, 2, respectively. Promoter regions of *cpxP*, *rpoE* and *rprA*, selected as representative targets of the Cpx, σ^E^ and Rcs regulons, respectively, were amplified by PCR using DNA from MG1655 as the template and oligonucleotide pairs AP797-AP798, for *cpxP* promoter, AP793-AP794, for *rpoE* promoter, and AP791-AP792, for *rprA* promoter. The PCR products were then digested with *EcoR*I and *BamH*I and cloned into the corresponding sites of pRS415 promoter probe vector. The inserts were verified by sequencing.

**Table 2:** Oligonucleotides.

Name	Sequence^a^ (5′ to 3′)	Description
AP791	gagag**gaattc**AATTGATATTTGCTTGCTCTTCC	pRS415-*rprAp* construction; *EcoR*I
AP792	attgt**ggatcc**GAGCTAATAGTAGGCATACGGAC	pRS415-*rprAp* construction; *BamH*I
AP793	gagag**gaattc**CAGCATGACAAACAAAAAC	pRS415-*rpoEp* construction; *EcoR*I
AP794	attgt**ggatcc**TTAGCTGCAATTTGAGCAAGC	pRS415-*rpoEp* construction; *BamH*I
AP797	gagag**gaattc**AGACGTCGCTAATCCATGAC	pRS415-*cpxPp* construction; *EcoR*I
AP798	attgt**ggatcc**TCT TTC TGT CGC GAT TCA ACG	pRS415-*cpxPp* construction; *BamH*I

^a^*E. coli* genomic sequence in uppercase; restriction sites in bold lowercase.

### 2.3 RNA sample preparation

Bacterial cultures were grown in 100 mL LB medium at 37 °C with shaking at 180 rpm until the optical density at 600 nm (OD_600_) reached 0.2. Cultures were then diluted to prepare secondary cultures in 25 mL LB medium with a starting OD_600_ of 0.1, either in the absence or presence of thanatin at a final concentration of 5.25 μg/mL (1.5 × MIC). We performed this dilution to match the growth phase used in our previous characterization of thanatin-induced LptA degradation kinetics (Moura et al., 2020), ensuring the transcriptomic timepoints reported here are directly comparable to the molecular timeline already established for this system. Samples for RNA extraction were collected at time 0 (before dilution) and after 5, 30, and 120 min by harvesting 10, 5, and 2 mL of culture, respectively, followed by immediate stabilization in pre-chilled (−80 °C) ethanol:acetone (1:1; 40, 20, and 10 mL, respectively) and immediately frozen in liquid nitrogen and stored at −80 °C until they were processed for total RNA isolation (Renzoni et al., 2006). Total RNA was extracted using the RNeasy Plus Mini Kit (Qiagen) as previously described (Roch et al., 2019). The experiment was performed in two biological replicates.

### 2.4 RNA-seq library preparation and sequencing

Total RNA concentration was determined using the Qubit fluorometer (Life Technologies), while RNA quality and integrity were evaluated with the Bioanalyzer system (Agilent Technologies). 1 μg of total RNA was subjected to ribosomal RNA depletion using Illumina’s bacterial Ribo-Zero kit. Sequencing libraries were subsequently generated with the Illumina TruSeq Stranded Total RNA Library Preparation Kit. Library concentration was quantified using Qubit, and library quality was verified on an Agilent TapeStation employing a DNA High sensitivity chip. Paired-end sequencing (150 × 150 bp) was performed on the Illumina HiSeqX v2.5 system. The quality of the sequencing data was assessed using FastQC (version 0.11.9).^1^ The reads were mapped onto the reference genome (NC_000913.3 for *E. coli* MG1655) using STAR (version 2.7.4a) command and alignment converted into BAM format with Samtools (version 1.16.1).

The number of reads overlapping each gene was counted with R package GenomicAlignments using method summarizeOverlaps (inter.feature = FALSE, mode = Union).

### 2.5 Statistical analysis of RNA sequencing data

RNA-seq analysis was performed with *n* = 2 biological replicates per condition using DESeq2 1.46. DESeq2 has been independently benchmarked as controlling its false discovery rate (≲5%) across replicate numbers including *n* = 2, with the dominant consequence of reduced replication being lower sensitivity (or false negatives) rather than inflated false-positive calls, and is validated for use with as few as two replicates per condition when combined with a fold-change threshold (Schurch et al., 2016); the threshold applied in this study (|log2FoldChange| ≥ 1) exceeds the validated lower bound (*T* ≥ 0.5). To address this loss of sensitivity directly, we applied DESeq2’s built-in equivalence test genome-wide at each of the three matched timepoints (see Supplementary Table S1 for implementation details). Rather than testing for any deviation from zero, this formally evaluates whether a gene’s fold change lies below the reporting threshold, distinguishing genes that are genuinely unchanged from those for which an effect cannot be excluded with the available data. This identified 751 gene–timepoint comparisons for which a true effect reaching the reporting threshold cannot be ruled out; these are flagged in Supplementary Table S1. Principal component analysis (PCA) was performed to evaluate the clustering of experimental replicates. The PCA analysis revealed that the replicates at 5, 30, and 120 min after treatment clustered tightly, indicating a high degree of reproducibility between replicates (Supplementary Figure S1).

### 2.6 Functional enrichment analysis

To identify functional relationships among differentially expressed genes (DEGs) showing a ≥1-fold change in expression (log_2_FC ≥ 1) upon treatment with thanatin across different time points, we performed functional annotation clustering using KEGG (Kyoto Encyclopedia of Genes and Genomes). Differential expression analysis was conducted using DESeq2 in R. Gene Ontology (GO), KEGG, and EcoCyc enrichment analyses were performed using Fisher’s exact test, and all *p* values were adjusted using the Benjamini–Hochberg procedure to control the false discovery rate (FDR). The *E. coli* K-12 annotation database used for mapping was *org. EcK12.eg.db*.

### 2.7 β-Galactosidase assays

Cultures of BW25113 transformed with reporter-probe vectors were grown in 35 mL of LB medium at 37 °C with shaking at 180 rpm until the OD_600_ reached 0.2. Thanatin treatment was performed as described above, and samples from treated and untreated cultures were collected by centrifugation after 5, 30, and 120 min (5, 4, and 1 mL, respectively). Cell pellets were resuspended in 1 mL of 0.9% NaCl solution and permeabilized by addition of 25 μL of 0.1% SDS and 50 μL of chloroform, followed by mixing for 10 s and incubation for 2 min at room temperature. Appropriate volumes of the permeabilized cell suspensions were used to measure β-galactosidase activity according to the method of Miller (Miller, 1992).

Briefly, 0.05–0.1 mL of permeabilized cells was added to Z buffer (final volume 1 mL) supplemented with 100 mM β-mercaptoethanol and incubated at 28 °C for 5 min. A blank sample without cells was included as a control. The reaction was initiated by adding 200 μL of 0.4% *o*-nitrophenyl-β-D-galactopyranoside (ONPG), pre-equilibrated at 28 °C, and terminated after 1 min by addition of 500 μL of 1 M Na_2_CO_3_. Absorbance was measured at 420 nm and 550 nm using a plate reader (EnSpire Multimode Plate Reader, PerkinElmer).

Optical density at 600, 420 and 550 nm were used to calculate Miller units. Experiments were performed with five biological replicates and two technical replicates.

Fold-change relative to the untreated control was calculated per replicate at each time point. Data are presented as the mean ± standard error of the mean (SEM). To test whether reporter activation was significant at each timepoint, one-sample *t*-tests were performed against a null fold-change of 1, with Benjamini-Hochberg correction applied within each reporter across the three timepoints. To assess whether activation increased over time, log_2_-transformed fold-changes were analyzed by one-way repeated-measures ANOVA with post-hoc pairwise comparisons adjusted using Tukey’s honestly significant difference method. All analyses were performed in R version 4.5.3.

### 2.8 Colanic acid extraction and quantification

CA was extracted from BW25113 wild type and Δ*wcaJ* mutant cells by heat treatment followed by ethanol precipitation and quantified by measuring L-fucose content using the cysteine-sulfuric acid method (Dische and Shettles, 1951). BW25113 wild-type and Δ*wcaJ* cultures were treated with thanatin at 1.5 × MIC for 120 min as described at paragraph 2.3 prior to CA extraction, matching the conditions previously used to characterize CA induction under LPS transport blockade (Moura et al., 2020). Then, 10 mL of treated and untreated control cultures were incubated at 100 °C for 15 min to inactivate CA-degrading enzymes and promote polysaccharide release. Samples were cooled to room temperature and centrifuged at 13,000 × *g* for 15 min. The supernatants were collected, and three volumes of ice-cold anhydrous ethanol were added. Samples were incubated on ice for 4 h (or overnight) to allow polysaccharide precipitation. Following incubation, samples were centrifuged at 10,000 × *g* for 15 min, the supernatants were discarded, and the resulting pellets were air-dried to remove residual ethanol. Pellets were resuspended in 1 mL of distilled water.

For CA quantification, 10–100 μL of the CA preparation was diluted to 1 mL with distilled water and mixed with 4.5 mL of 18 MH_2_SO_4_/H_2_O (6:1, v/v). Samples were heated at 100 °C for 20 min, cooled to room temperature, and absorbance was measured at 396 and 427 nm prior to cysteine addition (A_396_-co and A_427_-co). Subsequently, 100 μL of 3% cysteine hydrochloride was added, and after at least 1 h, absorbance was measured again at 396 and 427 nm (A_396_-cy and A_427_-cy). Corrected absorbance values were obtained by subtracting control readings from cysteine-treated samples, and the difference (A_396_–A_427_) was calculated. L-fucose concentration was determined using a standard curve generated with L-fucose (5–100 μg/mL). CA levels were normalized to cell density measured as OD_600_.

### 2.9 Determination of steady-state protein levels

DegP and OmpF steady-state levels were assessed in MG1655 strain by Western blot. Bacterial cultures were treated with thanatin 1.5 × MIC as described previously. Whole cell extracts for protein analysis were collected and harvested by centrifugation (5,000 × g, 10 min) at time 0 and 30 and 120 min after thanatin treatment. The cell pellets were resuspended in a volume (in ml) of SDS Laemmli buffer equal to 1/24 of the OD_600_ of the sample. The samples were boiled for 5 min and equal volumes (15 μL) were separated by 12.5% SDS-PAGE. Proteins were transferred onto nitrocellulose membranes (GE Healthcare), and immunoblotting was performed using polyclonal antibodies raised in rabbit against DegP (CusaBio) and OmpF (Cusabio) as primary antibody at dilutions of 1:3000 and 1:1000, respectively. As secondary antibody, goat anti-rabbit immunoglobulin (Li-Cor) was used at a dilution of 1:15,000. Bands were visualized by an Odyssey Fc imaging system (Li-Cor GmbH).

The steady-state levels of LptA and LptB shown in Supplementary Figure S3 were assessed by Western blotting using primary antibodies raised in rabbit against LptA (GenScript Corporation), and LptB (kindly provided by Dr. N. Ruiz, Department of Microbiology, The Ohio State University, Columbus, OH, USA) at dilutions of 1:1,000, and 1:10,000, respectively.

### 2.10 Motility assays

Overnight cultures of different MG1655 strains were grown in LB and inoculated with a sterile toothpick on swarming plates with or without thanatin. The swarming motility plates were prepared with 0.5% Difco agar, 1.0% Difco Bacto-tryptone, 0.5% Difco Yeast Extract, 0.5% NaCl and 0.5% D-(+)-glucose with and without thanatin. A concentration of 0.58 μg/mL (corresponding to 0.16 × MIC) was used as determined from preliminary experiments aimed at identifying the optimal thanatin concentration that preserves bacterial cell survival while maintaining an antibiotic effect. The plates were dried for 5–6 h at room temperature before being inoculated and were photographed after a 24 and 48 h incubation at 37 °C.

### 2.11 Imaging analysis

A single colony of *E. coli* MG1655 and MG1655∆*cpxR* were inoculated into LB medium and grown overnight at 37 °C. The following day, secondary cultures were inoculated at an initial OD₆₀₀ of 0.05 in 20 mL LB and grown at 37 °C to an OD_600_ of 0.2. Cultures were then diluted 1:2 into two 10 mL aliquots (treated and untreated). The treated samples were exposed to 1.5 × MIC thanatin, while an equivalent volume of sterile water was added to the untreated controls.

Cultures were incubated for up to 120 min, and samples were collected at 30 and 120 min. At each time point, cells corresponding to a total OD_600_ of 3.5 were fixed with a solution containing 1 mL of 37% formaldehyde and 21 μL of 25% glutaraldehyde and incubated for 20 min at 37 °C. Cells were centrifuged at 4,000 × *g* for 10 min, resuspended in 1 mL PBS, and centrifuged again at maximum speed for 3 min. The final pellet was resuspended in 0.2 mL PBS.

Fixed cells were stained with 5 μg/mL FM5–95 membrane dye (ThermoFisher) and 1 μg/mL DAPI DNA dye (Sigma). 5 μL of cell suspension were deposited on microscope slides and sealed with a glass coverslip (VWR or Fisher Scientific). Slides were visualized using a Zeiss LSM 900 equipped with C Plan-Apochromat63x/1.4 Oil DIC (WD = 0.14 mm), PALM MicroLaser Systems, AxioCAM ICc1 camera and PALM ROBO-software.

## 3 Results

### 3.1 Transcriptome analysis of thanatin exposed *E. coli* cells

We previously showed that treatment of *E. coli* cultures with thanatin at 1.5 × MIC leads to growth arrest accompanied by a slight decrease in cell viability and progressive degradation of LptA, a marker of Lpt complex disassembly (Martorana et al., 2021; Moura et al., 2020). To gain insights into the mechanisms underlying the cellular response to thanatin, we sought to monitor global gene expression by performing transcriptome analyses. For this purpose, we used the *E. coli* K-12 MG1655 strain, as it is a well-characterized wild-type reference strain with a widely adopted genome annotation suitable for global regulation studies. We treated early log-phase cultures of MG1655 (OD_600_ = 0.1) with 5.25 μg/mL thanatin (corresponding to 1.5 × MIC) and then collected the RNA samples for transcriptomic profiling by RNA-seq from cells before (C0) and at 5, 30, and 120 min after thanatin exposure. In parallel, RNA samples were collected from the untreated culture at the same time points (Figure 1A). RNA-seq analysis was performed on two biological replicates. Data stability and reproducibility across replicates were evaluated using PCA (Supplementary Figure S1). To quantify replicate concordance, we compared the Euclidean distance between each pair of biological replicates with the distances separating samples from different experimental conditions, computed in PCA space using all principal components on rlog-transformed counts, which preserves distances in the underlying expression data. Within-replicate distances were 6.3-fold smaller than between-condition distances (median 9.0 vs. 57.1). For 5 of 7 conditions, each sample’s nearest neighbor was its own biological replicate. The exceptions were the 5-min treated and untreated samples which were mutually nearest neighbors, consistent with limited transcriptional divergence at this early time point, before treated and control samples diverged progressively over the time course. The untreated 0-min pair showed the largest within-replicate distance (30.7, vs. 5.5–12.4 for the remaining six conditions), although the two replicates remained closer to each other than to any other sample. At 0 min, no treatment or time effect is present, so this distance reflects residual biological variation between cultures alone, without the contribution of a shared condition effect that compresses within-pair distances at later time points.

**Figure 1:**
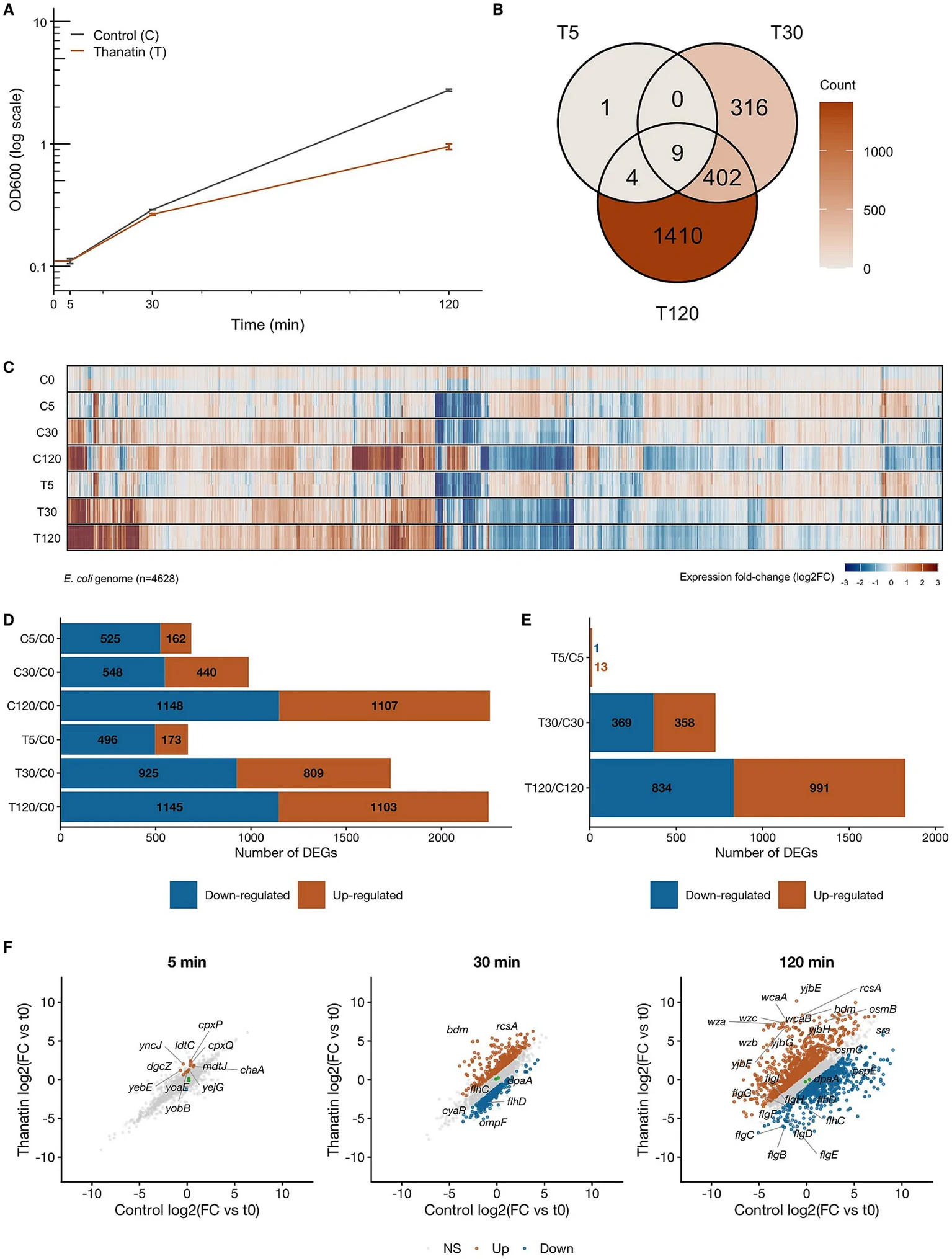
Transcriptomics of thanatin treated cells. **(A)** Timeline of thanatin exposure experiment. The culture of MG1655 was treated (T) or not (C) with 5.25 thanatin μg/mL (1.5 × MIC) and at following time points (0, 30 and 120 min) samples were collected for further analysis: time 0 (C0); early time point (T5, C5) and late time points (T30, C30 and T120, C120). Data points represent the mean of two independent replicates with standard deviation.**(B)** Venn diagram showing the number of common DEGs between control and thanatin-treated samples across different time points. **(C)** Heatmap showing the expression pattern of all *E. coli* genes. Shades of red and blue denote high and low relative expression, respectively, and white represents no expression change. **(D)** Number of differentially regulated genes (DEGs) with log2(FC) ≥ 1 and padj ≤ 0.05 relative to time 0 in control and thanatin-treated MG1655 cells. **(E)** Barplot showing number of upregulated and downregulated genes between treated and control samples at different time points. **(F)** Scatter plot for the time point comparisons, showing some of the interesting upregulated (red) and downregulated genes (blue) in thanatin-treated sample compared to control across different time points. Non-differentially expressed control housekeeping genes are shown in green.

To investigate the global expression profile, gene expression was quantified as Log_2_Fold Change (LFC) relative to time zero (C0) for each time point. As shown in the heatmap, the treatment induced a dramatic change in the expression profile at 30 and 120 min in treated cells (T30 and T120, respectively; late time points) compared to the untreated control samples (C30 and C120; Figure 1C). We applied a |LFC| ≥ 1 and adjusted *p* value < 0.05 selection criteria to identify the differentially expressed genes (DEGs) between treated and untreated samples relative to C0 and then directly comparing the three time points (Supplementary Table S1). Interestingly, the comparison across different time points between treated and control conditions revealed 9 common upregulated genes among 5, 30, and 120 min, whereas 4 upregulated DEGs were shared between 5 and 120 min (Figure 1B; Supplementary Table S1).

As summarized in Figure 1D, the number of DEGs relative to C0 was similar after 5 min in thanatin-treated (T5) and untreated (C5) samples (669 vs. 687), corresponding to 173 vs. 162 upregulated and 496 vs. 525 downregulated genes. On the contrary, the number of DEGs at 30 min in thanatin-treated sample (T30) was significantly higher (809 vs. 440 upregulated, and 925 vs. 548 downregulated) compared to C30. At 120 min, an approximately equal number of upregulated and downregulated DEGs were found in thanatin-treated sample compared to control condition (1,103 vs. 1,107 upregulated and 1,145 vs. 1,148 downregulated).

Direct comparison of gene expression between treated and untreated cells at each time point using the same selection criteria revealed a total of 14 DEGs (5 min), 727 DEGs (30 min), and 1825 DEGs (120 min; Figure 1E). These DEG populations are specific changes induced by thanatin treatment and account for 0.3, 15.7, and 39.4% of the genome of *E. coli* MG1655 strain, respectively.

Out of the early 14 DEGs at 5 min, 13 genes were upregulated and only 1 gene was downregulated in thanatin-treated cells. Similarly, for the late response time points, 358 genes were upregulated at 30 min and 369 genes were downregulated, whereas at 120 min 991 genes were upregulated and 834 genes were downregulated (Figure 1E). Some of the interesting upregulated and downregulated genes in thanatin-treated sample compared to control across different time points are shown in scatter plots (Figure 1F).

We verified stability of two housekeeping genes from orthogonal pathways: uronate utilization regulator (*uxuR*) and sulfur metabolism (*cysG*), both previously evaluated as candidate references in *E. coli* (Kruszewska-Naczk et al., 2024; Zhou et al., 2011). Both remained non-differentially expressed across all timepoints (|LFC| < 0.3, adjusted *p* > 0.1), confirming that the transcriptional changes observed at 120 min reflect specific regulatory responses (Figure 1F).

Globally, the DEGs were annotated using the Kyoto Encyclopedia of Genes and Genomes (KEGG) and Gene Ontology (GO) databases to identify the functional pathways (metabolic and signaling) associated with the enriched DEGs in the treatment conditions. The significant enrichment was determined by comparison to the annotation of reference transcripts using an adjusted *p* value <0.05.

Four broad functional categories were enriched upon thanatin treatment across the tested time points: cellular processes, environmental information processing, genetic information processing, and metabolism (Supplementary Figure S2). The most significant upregulated pathways at 30 min belonged to glycan biosynthesis, TCA cycle, exopolysaccharide biosynthesis and amino acid metabolism. The enriched downregulated functional categories corresponded to flagellar assembly pathway, ribosome and aminoacyl-tRNA biosynthesis.

The upregulated DEGs at 120 min included pathways associated with lipid metabolism, carbon metabolism, and energy metabolism, in addition to glycan biosynthesis, amino acid metabolism, and membrane transport. The downregulated DEGs at this time point belonged to cell motility, cellular community behavior (quorum sensing), phosphotransferase system (PTS). Other significant functional pathways related to downregulated genes were galactose metabolism, fructose and mannose metabolism, oxidative phosphorylation, and nitrogen cycle.

### 3.2 RegulonDB enrichment analysis to identify envelope stress regulons implicated in thanatin stress response

To investigate the relationship between enriched pathways and stress response systems, we performed a regulon-based enrichment analysis employing the RegulonDB (RegDB) database (Salgado et al., 2024). Applying the same selection criteria as described above, we identified regulon categories involved in the early (5 min) and late (30 and 120 min) response to thanatin treatment (Figure 2).

**Figure 2:**
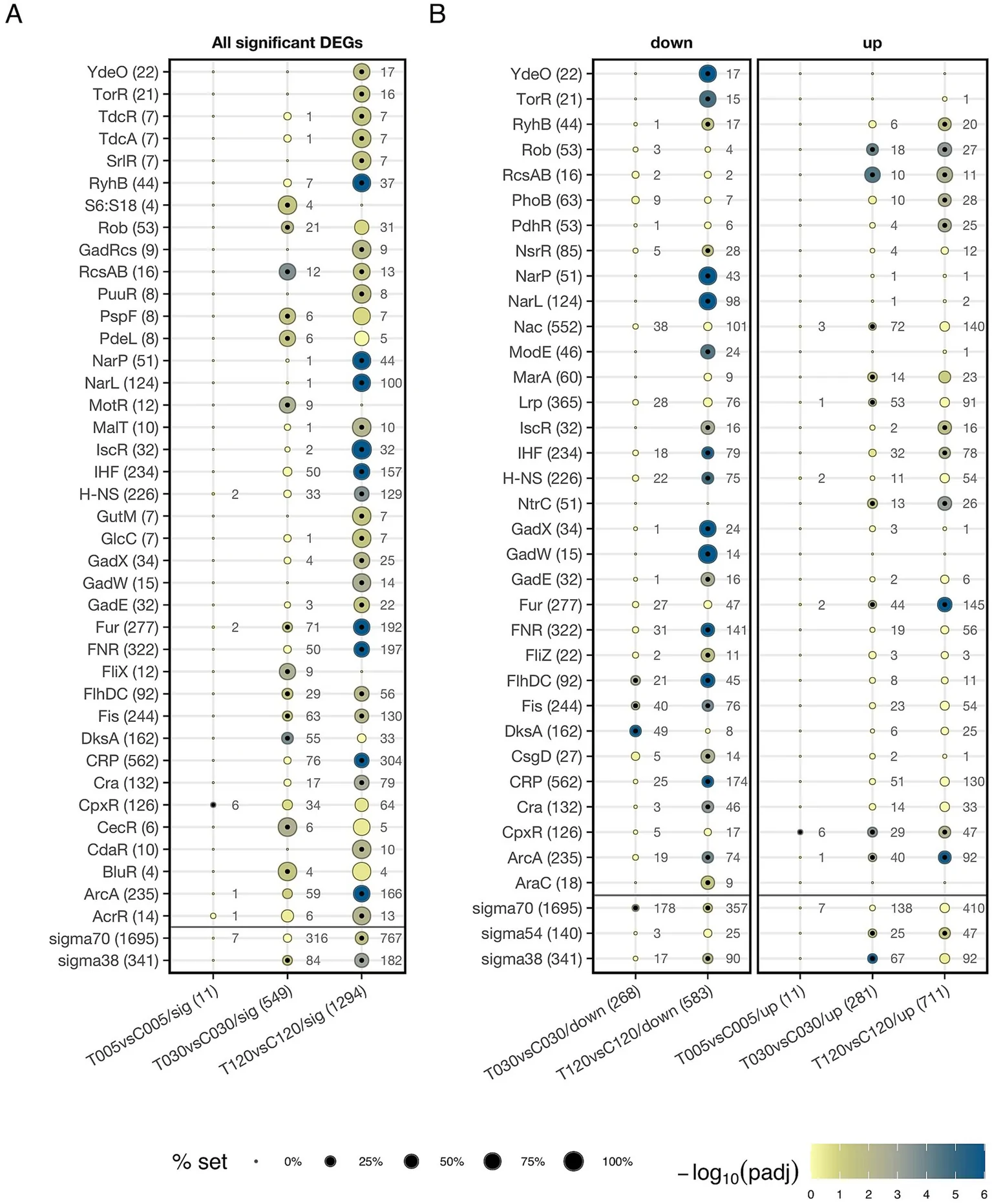
RegulonDB pathway enrichment analysis of significant DEGs across the three time points under thanatin treatment. **(A)** All significant (upregulated and downregulated) DEGs and **(B)** downregulated (down) and upregulated (up) enrichment analysis. The circle size represents the fraction of the number of DEGs regulated by the corresponding regulon out of the total number of DEGs mapping to the concomitant regulon. The color of the circle indicates the significance based on the Log_10_Padj < 0.05. More blue shade represents higher significance and more yellow represents low significance. The black dot in the middle of the circle means Log_10_Padj < 0.05.

The undirected regulon-based enrichment analysis was performed to identify transcriptional regulators whose regulons were over-represented among DEGs without considering whether targets were activated or repressed by the regulator. As shown in Figure 2A, the Cpx regulon (CpxR) was activated early (5 min) following thanatin treatment with highly significant differential expression of 6 genes out of the total 126 Cpx-regulated genes annotated in RegDB. Cpx activation continued over time, as 34 genes were differentially expressed in the treated condition compared to the control at 30 min (Figure 2A). Collectively the undirected enrichment analysis revealed a time-dependent enrichment of multiple regulons (dominated by envelope stress and motility regulators).

Directed regulon-based enrichment analysis revealed that the majority of Cpx-regulated DEGs were consistently upregulated, with stronger enrichment at later timepoints (29 and 47 DEGs were significantly upregulated at 30 and 120 min, respectively; Figure 2B). Interestingly, RcsAB was found to be the most significantly enriched regulon at 30 min, with 12 DEGs out of 16 showing differential expression (10 and 11 upregulated genes at 30 and 120 min, respectively). FlhDC regulon controlling cell motility was found to be enriched at 30 min, with 29 DEGs in sample T30 out of 92 genes (Figure 2A), among which 21 genes showed downregulation (Figure 2B).

A large number of genes were differentially expressed at the longest tested time point (120 min), which corresponds to complete depletion of LptA (Supplementary Figure S3) and a marked reduction in growth rate (Figure 1A); we reasoned that the observed transcriptional changes at this stage likely reflect secondary effects resulting from impaired LPS transport. To avoid confounding effects associated with this late-stage condition, we therefore focused our analysis on the 5- and 30-min time points, which are more likely to capture early and primary cellular responses to thanatin-induced damage. The 120 min time point will not be discussed as an independent condition, but rather considered in the context of transcriptional changes that emerged at 30 min and persisted at 120 min. All reported genes met a threshold of padj < 0.05, and unless otherwise stated, gene regulation is discussed in the context of comparison with the matched time point untreated control sample.

### 3.3 Early response to thanatin treatment

The list of the 14 DEGs in thanatin treated cells at 5 min compared to the control is reported in Table 3. Amongst these early DEGs, 6 genes (namely *dgcZ*, *ldtC*, *yebE*, *cpxP*, *cpxQ* and *chaA*) belong to the Cpx regulon. Five of these genes are among the 9 common genes that are upregulated across all time points during thanatin treatment (Figure 1B), together with *yobB*, *ynfD* and *yejG*. No genes belonging to other ESRs were differentially expressed at the 5 min treated time point (Figure 2). Consistent with this, among the DEGs showing increased expression at this early time point were regulators of the Cpx system, namely *cpxQ* and *cpxP*. *cpxQ* is a sRNA originating from the 3′ UTR region of *cpxP*. While the latter encodes for a regulator involved in a negative feedback loop of the system (Mitchell and Silhavy, 2019), the former is a negative regulator of the periplasmic chaperone Skp. Downregulation of *skp* has been shown to prevent insertion of OMPs in the IM, thereby preserving membrane homeostasis (Grabowicz et al., 2016). Lower abundance of Skp has been previously observed by proteomic analysis in *lptC*-depleted cells experiencing LPS transport disruption (Martorana et al., 2014). While no significant downregulation of Skp expression was observed at 5 min in our transcriptomic analysis, a mild but statistically significant decrease emerged at 30 and 120 min (LFC −0.54 and −0.59, respectively), in agreement with prior studies.

**Table 3:** Early differentially expressed genes.

Gene	Function	Category	log_2_FC_5 min^a^
***yncJ***	DUF2554 domain-containing putative exported protein		2.4
***cpxP*** ^*^	Negative regulator of the Cpx pathway	Stress signal	2.1
***ldtC*** ^*^	L, D-transpeptidase	PG remodelling	2.0
***yebE*** ^*^	Unknown (IM)		1.9
***cpxQ*** ^*^	sRNA regulator of CpxP	Stress signal	1.7
***dgcZ*** ^*^	Diguanylate cyclase	Motility	1.6
*mdtJ*	multidrug/spermidine efflux transporter	Membrane transport	1.2
chaA^*^	Na+/K+ proton antiporter-growth at alkaline pH	Membrane transport	1.1
***yobB***	Putative carbon-nitrogen hydrolase	Metabolism	1.1
***ynfD***	Putative exported unknown protein		1.1
***yejG***	Unknown		1.1
*ydgV(mdtU)*	Unknown/operon *mdtUJI*		1.0
*yoaE*	TerC family IM protein	Membrane transport	1.0
*hokB*	Toxin	TA system	-1.2

^a^log_2_FoldChange with respect to the untreated control at the same time point relative to time 0. ^*^Genes belonging to CpxRA regulon. TA, toxin-antitoxin system. Genes upregulated in all three time points are bolded.

Most of the other DEGs identified at this early time point are poorly or uncharacterized, even if associated with antimicrobial resistance. The gene with the greatest fold-change increase was *yncJ*, which similarly to *ynfD* encodes a small putative exported protein with unknown function. Notably, *yncJ* upregulation depends on NlpE overexpression, a well-known Cpx-inducing signal (Raivio et al., 2013) and its upregulation has been documented in *E. coli* as a transcriptional response to the inhibition of the lipoprotein trafficking system (Lorenz et al., 2016). Strong upregulation was observed in *ldtC* at 5 min, which was maintained at 30 and 120 min. *ldtC* encodes an L, D-transpeptidase responsible for anchoring OM-localized Braun’s lipoprotein Lpp to the PG, thereby increasing cell envelope stability (Magnet et al., 2007).

Another gene whose expression was markedly increased compared to the untreated control was *dgcZ*, which encodes a diguanylate cyclase previously implicated in biofilm production (Lacanna et al., 2016).

Genes involved in membrane transport were also upregulated, including the efflux component *mdtJ*, the cation/proton antiporter *chaA*, and *yoaE*, which encodes an IM transporter previously implicated in antibacterial resistance in *Salmonella enterica* (Huang et al., 2020).

Increased expression of *hokB*, which encodes a toxin of a type I toxin-antitoxin system, has been associated with the induction of persister cells through membrane potential depolarization (Verstraeten et al., 2015). In our dataset, *hokB* was the only gene downregulated at 5 min.

Collectively, the genes induced at 5 min encode functions in OM-PG linkage (*ldtC*), multidrug efflux (*mdtJ*), IM cation homeostasis (*chaA*), and biofilm regulation (*dgcZ*).

### 3.4 Transcriptome changes induced upon 30 min-treatment with thanatin

Transcriptomic analysis revealed that thanatin treatment induced 727 DEGs at 30 min compared with untreated samples (Figure 1E). At this time point, the disassembly of the Lpt machinery and consequently the degradation of LptA is not yet detectable (Supplementary Figure S3). Since the cell envelope is a dual target for thanatin (LPS is the recognized moiety for peptide entry into the cell and the intracellular target of thanatin-mediated antibacterial activity; Sinha et al., 2022), we focused our analysis on envelope-associated stress response systems activated at 30 min following thanatin exposure, with the aim of identifying structural adaptations required to counteract its activity. Specifically, we examined the activation of the canonical ESRs—CpxRA, RcsAB/RcsB, PspF and σ^E^ (Figure 3)—together with the FlhDC motility regulon and the metabolic and lipid-homeostasis responses that accompany them.

**Figure 3:**
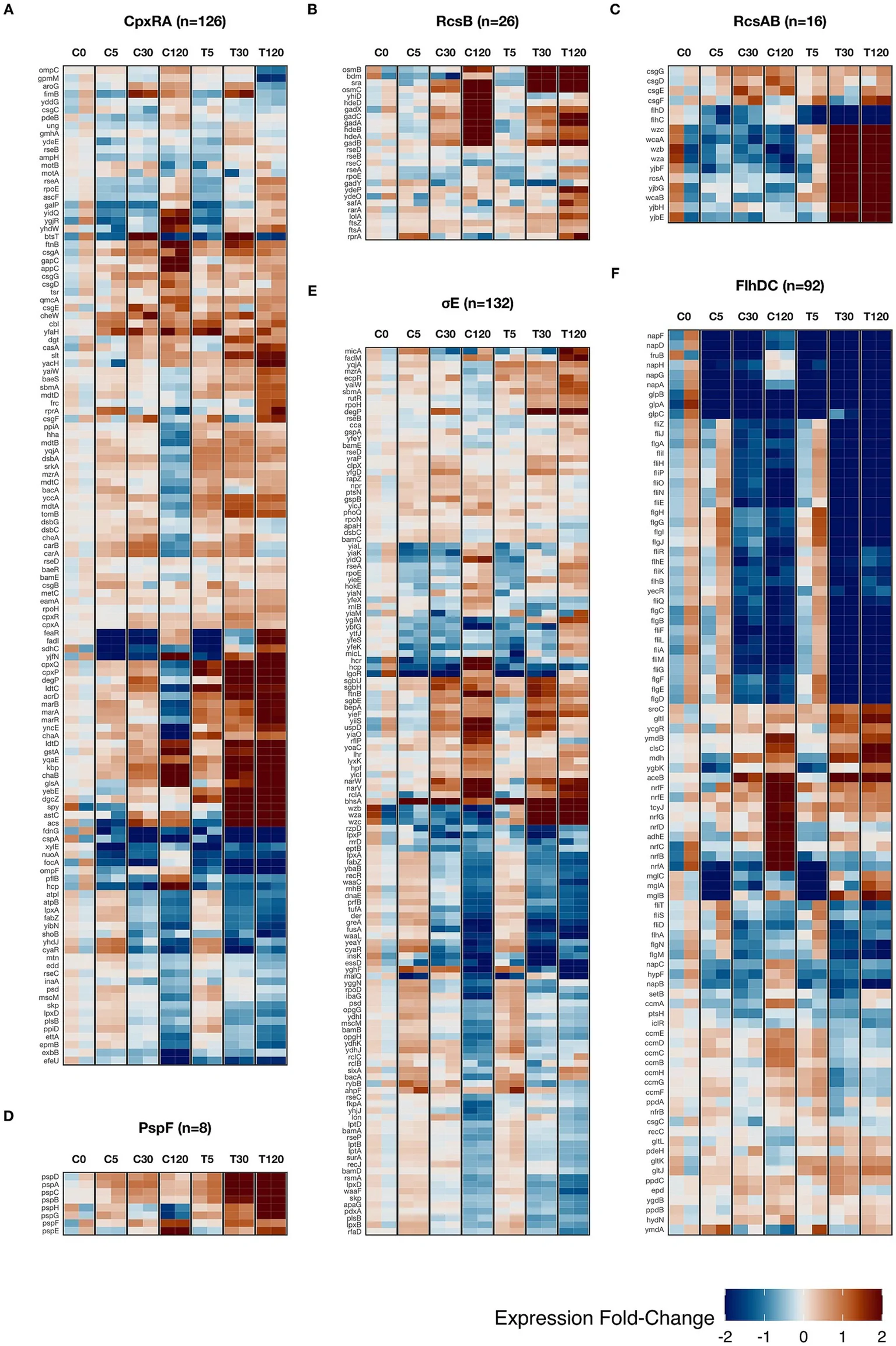
Heatmap showing differentially expressed genes belonging to the following regulon categories: **(A)** CpxRA, **(B)** RcsB, **(C)** RcsAB, **(D)** PspF, **(E)** SigmaE, and **(F)** FlhDC. Shades of red and blue denote high and low relative expression, respectively and white represents no expression change.

#### 3.4.1 Cpx stress response activation at late time points upon thanatin treatment

Transcriptomic analysis of *E. coli* MG1655 cells exposed to thanatin identified Cpx as the most enriched stress response system, with significant activation observed at early and late time points (Figure 3A). At 30 min, 34 Cpx-dependent DEGs were identified, encompassing functions related to resistance to cationic antimicrobials, envelope stress response, osmotic stress adaptation, protein quality control, regulation of phospholipid metabolism, and control of general OM porins (Table 4). Notably, six of these DEGs were already upregulated at 5 min (Table 3) and remained upregulated throughout all analyzed time points, underscoring their relevance in Cpx-mediated adaptative response. Several of the 30 min Cpx-dependent DEGs encode protein quality-control functions. The most strongly upregulated Cpx dependent gene was *spy,* which encodes an ATP-independent periplasmic chaperone that prevents protein aggregation and promotes protein refolding (Combs and Silhavy, 2024). Consistently, increased expression of the gene encoding the periplasmic chaperone-protease DegP and of *yjfN*, encoding for a specific activator of DegP (Kim et al., 2018), were also observed (Ge et al., 2014; Soltes et al., 2017). In addition, *cpxQ*-mediated repression of *skp* and direct downregulation of *ompF*, encoding one of the major nonspecific OM porins in *E. coli* (Nikaido, 1992), were also observed.

**Table 4:** CpxRA regulated genes differentially expressed at 30 min.

Gene	Function	Category	log_2_FC_30min^a^	log_2_FC_120min^a^
*spy*	ATP-independent periplasmic chaperone	Protein folding	5.1	7.2
*yjfN*	Protease activator	Protein folding	3.6	n.s.
dgcZ^*^	Diguanylate cyclase	Motility	3.5	5.3
yebE^*^	Unknown (IM)		3.3	5.4
*astC*	Succinylornithine transaminase	Metabolism	2.5	6.7
cpxP^*^	Negative regulator of the Cpx pathway	Stress response	2.4	5.7
*acs*	Acetyl-CoA synthetase (AMP-forming)	Metabolism	2.4	5.9
*acrD*	Multidrug efflux pump RND permease subunit	Drug efflux	2.1	3.3
cpxQ^*^	sRNA regulator of CpxP	Stress response	2.1	5.9
*fadI*	3-ketoacyl-CoA thiolase	β-oxidation of fatty acids	1.9	2.7
*ldtD*	L,D-transpeptidase	PG-remodelling	1.9	1.1
*yqaE*	Predicted membrane protein of unknown function		1.7	2.4
*sdhC*	Succinate:quinone oxidoreductase, membrane protein	Metabolism	1.7	2.7
*kbp(ygaU)*	K+ binding protein	PG-remodelling	1.6	n.s.
*tomB*	Hha toxicity modulator	TA system	1.5	2.3
*mdtA*	Multidrug efflux pump	Drug efflux	1.5	2.2
*feaR*	DNA-binding transcriptional activator	Degradation of toxic compounds	1.5	1.6
*degP*	Periplasmic serine endoprotease	Protein folding	1.4	4.8
*marR*	DNA-binding transcriptional repressor	Drug efflux	1.4	3.9
*slt*	Soluble lytic murein transglycosylase	PG-remodelling	1.4	1.3
*marA*	DNA-binding transcriptional dual regulator	Drug efflux	1.4	4.2
*gstA*	Glutathione S-transferase	Degradation of toxic compounds	1.3	n.s.
*marB*	Multiple antibiotic resistance protein	Drug efflux	1.2	4.5
*dgt*	dGTP triphosphohydrolase	Replication fidelity	1.1	n.s.
*chaB*	Putative cation transporter regulator	Drug efflux	1.0	0.5
*ftnB*	Putative ferritin-like protein	Unknown	1.1	−1.4
*mdtB*	Multidrug efflux pump	Drug efflux	1.0	2.1
ldtC^*^	L,D-transpeptidase	PG-remodelling	1.0	4.5
*glsA*	Glutaminase 1	Glutamate biosynthesis	1.0	−3.7
*efeU*	Inactive ferrous iron permease	Pseudogene	−1.4	2.5
*yhdJ*	DNA adenine methyltransferase	DNA modification	−1.5	1.1
*cspA*	Cold shock protein	Stress response	−1.8	n.s.
*cyaR*	Small regulatory RNA	Stress response	−2.0	n.s.
*ompF*	Outer membrane porin F	Transport	−3.4	−5.4

^a^log_2_FoldChange with respect to the untreated control at the same time point. ^*^Indicates genes already up regulated at 5 min. n.s., not significant. TA, toxin-antitoxin system.

Alongside protein quality control, PG remodelling became increasingly prominent at the 30 min treated time point, as evidenced by the upregulation of *ldtD* and *ldtC*. The *ldtD* gene encodes a L,D-transpeptidase that forms non-canonical 3-3 PG crosslinks and is activated in response to LPS-related perturbations (Morè et al., 2019; Magnet et al., 2008). As mentioned previously, *ldtC* encodes the L, D-transpeptidase that attaches the OM-localized lipoprotein Lpp to the PG (Magnet et al., 2007). In line with this observation, *dpaA* (formerly *ldtF*/*yafK*), which encodes a murein endopeptidase responsible for detaching the Lpp from PG (Winkle et al., 2021) was found to be strongly downregulated at 30 (LFC −1.24) and 120 min (LFC −1.19; Supplementary Table S1). This response matches previous data showing that impaired LPS transport induces a PG remodelling program to avoid cell death (Morè et al., 2019). The *lpt* genes themselves showed no such response: despite being the direct target of thanatin, none displayed altered mRNA levels at any time point. A formal equivalence test (see Material and Methods) confirmed that the fold-change for each of the seven *lpt* genes was statistically excluded from reaching the two-fold reporting threshold at every timepoint measured (Supplementary Table S1).

The gene encoding the lytic transglycosylase Slt was also upregulated at 30 and 120 min. Slt degrades non-crosslinked PG strands, thereby facilitating PG turnover (Cho et al., 2014). Interestingly, PG degradation products have been shown to function as activating signals for the Cpx system.

The genes *yqaE* and *kbp*, constituting a transcriptional unit, were also upregulated under thanatin treatment. Kbp is a K^+^-binding protein that interacts with the PG through a LysM domain and plays a role in PG remodelling during stress (Bernal-Cabas et al., 2015). It encodes a predicted membrane protein with unknown function, implicated in antibiotic resistance mechanisms (Raivio et al., 2013). Notably, *yqaE* expression is regulated post-transcriptionally by the sRNA *cyaR* (De Lay and Gottesman, 2009), which was highly downregulated in this condition—likely contributing to the observed upregulation of *yqaE*.

The *marRAB* operon, which encodes a regulatory system implicated in resistance to multiple antibiotics, including cationic antimicrobial peptides (CAMPs) (Warner and Levy, 2010), was significantly upregulated over time following thanatin treatment. MarA functions as an autoregulator, while MarR serves as an autorepressor which suppresses the expression of the operon. In addition to regulating the *marRAB* operon, MarA also controls the expression of other drug resistance genes (Ariza et al., 1994). Overexpression of *marA* has been shown to compensate for defects in lipopolysaccharide (LPS) production (Maniyeri et al., 2023).

Genes encoding multidrug efflux proteins, *mdtA*, *mdtB*, and *acrD* were highly expressed at 30 min. MdtA and MdtB are part of the *mdtABCD* operon, which encodes a resistance-nodulation-division (RND) type efflux pump and were also upregulated at 120 min (Table 4; Supplementary Table S1). MdtABCD efflux system is implicated in resistance to antibiotics, bile salts, and SDS (Nagakubo et al., 2002). AcrD, another member of the RND superfamily and component of the AcrAD-TolC efflux pump complex (Ma et al., 1994), is involved in resistance to a wide range of aminoglycoside antibiotics (Rosenberg et al., 2000). Among the most highly upregulated genes was *dgcZ*, which regulates biofilm formation and motility through c-di-GMP signaling (see above). Correspondingly, the toxicity regulator gene *tomB* of the *tom-hha* toxin-antitoxin (TA) system, the only TA system directly involved in biofilm formation (Ren et al., 2004), was already upregulated at 30 min. Indeed, the entire *tom-hha* system was significantly upregulated at the 120 min time point.

#### 3.4.2 Rcs system activation at late time points upon thanatin treatment

Transcriptomic analysis considered 16 RcsAB-dependent and 26 RcsB-dependent genes annotated from the RegulonDB database (Figures 3B,C). Transcription of genes belonging to the Rcs regulon are controlled by the RcsB response regulator, either as a homodimer or as a heterodimer with other response regulators, particularly RcsA (Mitchell and Silhavy, 2019). RcsAB-regulated genes are involved in CA biosynthesis and biofilm formation (Wall et al., 2018) and their expression was found to be activated upon thanatin treatment as a late response (30 and 120 min). Overall, among the RcsAB- and RcsB-dependent DEGs, 16 genes (mainly involved in exopolysaccharide synthesis) were already differentially expressed at 30 min (Table 5 and Figures 3B,C). *rcsA* was highly upregulated both at the 30 and 120 min time points. Genes involved in CA biosynthesis, including *wzc, wzb, wza, wcaA,* and *wcaB* genes were significantly upregulated (Stevenson et al., 1996; Figure 3B, Table 5). This observation is consistent with our previous studies demonstrating lipid A-core decoration with CA in cells defective for LPS export or treated with thanatin (Sperandeo et al., 2008; Moura et al., 2020). The gene *wzc* encodes a tyrosine kinase that negatively regulates CA biosynthesis, while the phosphatase *wzb* counteracts the negative regulation of *wzc* (Vincent et al., 2000). *wzc* and *wzb* form an operon with *wza*, a membrane polysaccharide export protein which showed marked upregulation at 30 and 120 min. *Wza* is likely involved in the transport of extracellular polysaccharides (Reid and Whitfield, 2005). In addition, the CA biosynthesis genes *wcaA* (a UDP-glucoronate glucurosyltransferase) and *wcaB* (an acetyltransferase) were also significantly upregulated.

**Table 5:** Rcs regulated genes differentially expressed at 30 min.

Gene	Function	Category	log_2_FC_30min^a^	log_2_FC_120min^a^
***bdm*** ^*^	Biofilm-dependent modulation protein	EPS production/Biofilm	7.5	8.0
*rcsA*	DNA-binding transcriptional activator	Stress response	5.9	8.8
wza^**^	Outer membrane polysaccharide export protein	CA production	5.9	10.6
wzc^**^	Protein-tyrosine kinase	CA production	5.3	9.7
***osmB***	Osmotically inducible lipoprotein	Stress response	5.3	5.6
wzb^**^	Protein-tyrosine phosphatase (activator)	CA production	5.2	10.9
wcaA^**^	UDP-glucuronosyltranferase	CA production	5.2	9.6
yjbE^***^	Exported unknown protein	EPS production/Biofilm	4.9	11.2
wcaB^**^	CA biosynthesis acetyltransferase	CA production	4.7	8.8
yjbF^***^	Lipoprotein, unknown	EPS production/Biofilm	3.8	9.1
yjbG^***^	GfcC family protein, unknown	EPS production/Biofilm	3.5	8.9
***sra*** ^*^	Ribosome-associated protein	Antibiotic tolerance?	3.1	1.6
***osmC***	Osmotically inducible peroxiredoxin	Stress response	2.3	2.3
yjbH^***^	Lipoprotein, unknown	EPS production/Biofilm	2.0	6.5
flhC^****^	DNA-binding transcriptional dual regulator	Motility	−1.7	−3.5
flhD^****^	DNA-binding transcriptional dual regulator	Motility	−1.9	−3.3

^a^log_2_FoldChange with respect to the untreated control at the same time point. CA, colanic acid. EPS, exopolysaccharide. ^*^, ^**^, ^***^ and ^****^ indicate genes belonging to the same transcriptional unit. Genes regulated by RcsB homodimer or heterodimer with other response regulators than RcsA are bold.

Interestingly, expression of the uncharacterized *yjbEFGH* operon was positively regulated at both 30 and 120 min. The operon is involved in the synthesis of extracellular polysaccharides (EPS) distinct from CA, and the induction of the operon was shown to be dependent on cell density and oxygen concentration (Ionescu et al., 2008). Rcs-mediated production of exopolysaccharides (CA and EPS) has previously been implicated in protection against serum-mediated complement killing in extraintestinal *E. coli*, underscoring the role of CA in preventing cell envelope damage (Miajlovic et al., 2014). Activation of the RcsAB heterodimer is also known to repress the *flhDC* operon, which encodes the master regulator of flagellar assembly and flagellin biosynthesis (Wall et al., 2018). Modulation of flagellar gene expression through alteration in translational fidelity has been described as a mechanism to improve bacterial fitness in response to environmental cues (Fan et al., 2016). Consistent with this model, the *flhDC* operon was downregulated at the 30 and 120 min time points compared with their matched controls.

Among the RcsA-independent DEGs, osmotic stress-related *osmB* and *osmC* genes, encoding for an IM lipoprotein and a peroxiredoxin, respectively, were markedly upregulated at both 30 and 120 min (Figure 3B). These proteins are typically induced under hyperosmotic conditions and during the stationary phase (Bouvier et al., 1998; Jung et al., 1990). A small-molecule inhibitor of *lolCDE* elicits a similar increase in *osmB* expression, as does polymyxin B (PMB) (Lorenz et al., 2016). In addition, thanatin treatment upregulated the biofilm-associated *bdm* gene (Francez-Charlot et al., 2005), along with its co-transcribed *sra* gene, which encodes a 30S ribosome-associated protein. Bdm has been implicated in osmotic-induced biofilm formation and in the reduction of motility through regulation of flagellar biosynthesis (Francez-Charlot et al., 2003).

#### 3.4.3 Effect of thanatin on the Psp, σ^E^ and FlhDC stress response systems

The phage shock protein (Psp) regulon system responds to stresses that compromise IM integrity and disrupt the proton motive force (PMF) (Delhaye et al., 2019). The transcriptional regulator of this system, *pspF*, functions as an enhancer-binding protein that stimulates σ^54^ (σ^N^)-dependent transcription. Although our transcriptomic analysis did not reveal significant expression of *pspF* at any stage of thanatin treatment, *pspABCD* genes were significantly upregulated at the 30 and 120 min time points. In our dataset, *pspE* was not detected at 30 min and was downregulated at 120 min (Table 6; Figure 3D). However, despite *pspABCDE* forming a single transcriptional unit, it is possible that *pspE* expression follows a different regulatory pattern to the other operonic genes.

**Table 6:** Stress response-regulated genes differentially expressed at 30 min.

Gene	Function	Category	log_2_FC_30min^a^	log_2_FC_120min^a^
Psp system				
*pspH*	ncRNA	Transcriptional regulation	1.7	4.7
*pspC*	Phage shock protein C/toxin of a putative PspC-PspB toxin-antitoxin pair	Stress response/TA	1.6	3.5
*pspB*	Phage shock protein B/antitoxin of a putative PspC-PspB toxin-antitoxin pair	Stress response/TA	1.5	3.6
*pspD*	Phage shock protein D/peripheral IM protein	Stress response	1.5	3.6
*pspG*	Phage shock protein G/IM protein	Stress response	1.5	4.8
*pspA*	Phage shock protein A/peripheral IM protein	Stress response	1.4	3.9
FlhDC				
*glpC*	Anaerobic glycerol-3-phosphate dehydrogenase subunit	Anaerobic respiration	1.7	−0.9
*mglB*	D-galactose/methyl-galactoside ABC transporter periplasmic binding protein	Transport/Chemotaxis	1.6	1.2
*aceB*	Malate synthase A	Metabolism	1.5	3.1
*gltI*	Glutamate/aspartate ABC transporter periplasmic binding protein	Transport	1.4	1.3
*clsC*	Cardiolipin synthase C	Lipid synthesis	1.2	0.9
*mglA*	D-galactose/methyl-galactoside ABC transporter ATP binding subunit	Transport	1.2	1.9
*mglC*	D-galactose/methyl-galactoside ABC transporter membrane subunit	Transport	1.1	2.0
*sroC*	Small regulatory RNA	Transcriptional regulation	1.1	1.2
*flhB*	Flagellar biosynthesis protein	Chemotaxis	−1.3	0.1
*fliH*	Flagellar biosynthesis protein	Chemotaxis	−1.3	−1.2
*fliQ*	Flagellar biosynthesis protein	Chemotaxis	−1.4	0.8
*flgI*	Flagellar P-ring protein	Motility	−1.6	−1.2
*fliO*	Flagellar biosynthesis protein	Chemotaxis	−1.7	−0.8
*fliP*	Flagellar biosynthesis protein	Chemotaxis	−1.8	0.2
*fliN*	Flagellar biosynthesis protein	Chemotaxis	−1.8	−1.1
*flgG*	Flagellar basal-body rod protein	Motility	−1.9	−1.4
*fliF*	Flagellar basal-body MS-ring and collar protein	Motility	−1.9	−2.4
*fliM*	Flagellar biosynthesis protein	Chemotaxis	−1.9	−1.5
*fliI*	Flagellar export ATPase	Chemotaxis/motility	−2.0	−1.1
*flgE*	Fagellar hook protein	Motility	−2.0	−2.9
*fliL*	Flagellar protein	Chemotaxis/motility	−2.1	−2.1
*flgJ*	Putative peptidoglycan hydrolase	Chemotaxis/motility	−2.1	−0.4
*fliA*	RNA polymerase sigma factor	Transcriptional regulation	−2.2	−2.0
*flgH*	Flagellar L-ring protein	Motility	−2.2	−1.5
*flgD*	Flagellar biosynthesis, initiation of hook assembly	Motility	−2.2	−3.2
*flgF*	Flagellar basal-body rod protein	Motility	−2.3	−2.4
*fliG*	Flagellar motor switch protein	Chemotaxis/motility	−2.4	−1.6
*flgB*	Flagellar basal-body rod protein	Motility	−2.6	−3.8
*flgC*	Flagellar basal-body rod protein	Motility	−2.7	−3.5
SigmaE regulon				
*wza*	Outer membrane polysaccharide export protein	CA production	5.9	10.6
*wzc*	Protein-tyrosine kinase	CA production	5.3	9.7
*wzb*	Protein-tyrosine phosphatase (activator)	CA production	5.2	10.9
*degP*	Periplasmic serine endoprotease	Protein folding	1.4	4.8
*ygiM*	Putative signal transduction protein	Unknown	1.4	3.3
*ftnB*	Putative ferritin-like protein	Unknown	1.1	−1.4
*gspB*	Type II secretion system protein	Unknown	−1.0	−0.3
*ydhK*	Putative transporter	Unknown	−1.0	1.0
*der*	50S ribosomal subunit stability factor	Ribosomal assembly	−1.2	0.5
*dnaE*	DNA polymerase III subunit α	DNA replication	−1.2	0.0
*rnhB*	RNase HII	DNA replication	−1.3	−0.1
*waaL*	O-antigen ligase	LPS biosynthesis	−1.4	−0.8
*yeaY*	Slp family lipoprotein	Unknown	−1.5	1.5
*rybB*	Small regulatory RNA	Stress response	−1.6	−0.5
*greA*	Transcription elongation factor	DNA transcription	−1.7	1.1
*cyaR*	Small regulatory RNA	Stress response	−2.0	0.7
*yghF*	Putative type II secretion system C-type protein	Protein transport	−2.6	2.1

^a^log_2_FoldChange with respect to the untreated control at the same time point. TA, toxin-antitoxin system.

Beyond its role in sequestering PspF to maintain system inactivity, free PspA has been reported to prevent proton leakage across the IM (Kobayashi et al., 2007). In addition, expression of *pspG* was upregulated at 30 min. PspG, an IM protein co-regulated with the *psp* operon, has been shown to negatively affect motility behavior in *E. coli* (Lloyd et al., 2004). Accordingly, upregulation of *pspH* was also observed. *pspH* is a small regulatory RNA encoded within the 3′ untranslated region of *pspG* mRNA and acts as a molecular sponge by base-paring the metabolic regulatory RNA Spot 42 reducing its cellular level (Papenfort and Storz, 2024). Decreased level of Spot 42 likely reflects a strategy adopted by the cells to coordinate envelope stress to carbon utilization.

The σ^E^ regulon was broadly downregulated after 30 min of thanatin exposure (Table 6; Figure 3E). Of the 17 σ^E^-regulated genes meeting the differential-expression threshold, 11 were downregulated and 6 upregulated, the latter dominated by three genes with the largest fold-changes in the set (*wza*, *wzb*, *wzc*), more plausibly RcsAB-driven CA-biosynthesis genes than direct σ^E^ targets. At the late time point, *ygiM* (encoding an IM-anchored protein of unknown function) was the only upregulated gene under σ^E^ regulation—aside from the CA-production genes *wza, wzb, wzc* (likely driven by RcsAB) and the Cpx-regulated *degP* and *ftnB* genes (see above).

As previously mentioned, thanatin treatment had a strong impact on *flhDC* operon (Table 6; Figure 3F). In line with this observation, in our transcriptomic analysis, the entire *flg* operon (*flgB*, *flgC*, *flgD*, *flgE*, *flgF*, *flgG*, *flgH*, *flgI*, and *flgJ*) was downregulated at the 30 and 120 min time points. These genes encode structural components of the flagellum, with the exception of *flgJ*, which encodes a periplasmic hydrolase required for rod assembly (Herlihey et al., 2014). The *fli* operon, which is also involved in flagellar assembly, exhibited a similar pattern of downregulation following thanatin treatment.

#### 3.4.4 Metabolic and lipid-homeostasis responses accompanying thanatin treatment

Beyond the canonical envelope stress regulons, thanatin treatment altered three further groups of genes: those governing LPS modification and cardiolipin synthesis, those mediating nutrient uptake, and those of fatty-acid β-oxidation and phospholipid transport. Among the upregulated DEGs, several genes are implicated in repression of LPS/envelope modification. The small RNA *sroC*, which is highly expressed during stationary phase and functions as a molecular sponge for other sRNAs (Lalaouna et al., 2019), was highly upregulated at both 30 and 120 min (Supplementary Table S1). Notably, in *Salmonella*, *sroC* post-transcriptionally regulates the activity of *mgrR* (also upregulated in our analysis; Supplementary Table S1), an sRNA involved in cell envelope modification. By binding *mgrR*, *sroC* alleviates repression of *eptB*, a gene encoding a phosphoethanolamine transferase that modifies LPS and reduces its net negative charge, thereby decreasing susceptibility to PMB (Acuña et al., 2016). In our analysis *eptB* was not upregulated (Supplementary Table S1). Interestingly, *clsC*, which encodes one of the three synthases responsible for cardiolipin synthesis in *E. coli* during stationary phase (Tan et al., 2012), was among the upregulated genes. Cardiolipin abundance in the IM has been implicated in regulation of LPS biogenesis (Douglass et al., 2021), therefore increased cardiolipin synthesis upon thanatin treatment may contribute to maintain OM homeostasis.

Genes belonging to the *mglABC* operon, responsible for galactose/glucose import in *E. coli* (Wu and Mandrand-Berthelot, 1995) were upregulated at 30 min. *mglC* encodes the integral membrane component of the D-galactose and D-galactoside ABC transporter, whereas *mglA* and *mglB* encode for the ATP-binding and periplasmic binding subunit of a D-galactose/methyl-D-galactoside ABC transport system, respectively. In line with this observation, the *gltIJKL* operon was also upregulated, with *gltI* (encoding the periplasmic protein of the glutamate/aspartate ABC transporter) showing significant upregulation at 30 and 120 min.

Transcriptomic profiling also revealed a coordinated induction of the fatty-acid β-oxidation (*fad*) regulon. Outer leaflet phospholipid accumulation in the OM triggers PldA-mediated phospholipid hydrolysis, generating free fatty acids (FFAs) that are converted by FadD to acyl-CoAs. Acyl-CoA accumulation has two parallel downstream consequences: stabilization of the LPS biosynthesis enzyme LpxC via a FadD-dependent, β-oxidation independent signaling route (May and Silhavy, 2018), and titration of the FadR repressor away from its target promoters, derepressing the β-oxidation genes *fadABEIJHM* (DiRusso et al., 1993; Fujita et al., 2007). The induction of this *fad* regulon subset at 30 and 120 min (*fadI* is annotated as a Cpx-dependent gene, see Table 4) is consistent with this signaling cascade (Supplementary Figure S4). Components of the lysophospholipid (LPL) recycling system downstream of PldA activity, *lplT* and *aas* (Enverso and Trent, 2026), were not differentially expressed at any time point (Supplementary Table S1). The *mla* operon (*mlaA-F*) was induced at 120 min, consistent with proteomic evidence of increased MlaD and MlaE abundance under LptC depletion (Martorana et al., 2014).

### 3.5 Phenotypic characterization of thanatin treated cells

In the transcriptomic dataset, Cpx- and Rcs-regulated genes accounted for the largest enriched regulons, whereas σ^E^-regulated genes were predominantly downregulated. Notably, Cpx is a key system for the early response to thanatin with 6 genes belonging to this regulon activated at 5 min. We therefore sought to monitor the activation of these regulons by assaying *lacZ* expression from *rpoE* (*rpoEp*), *cpxP* (*cpxPp*) and *rprA* (*rprAp*) promoters fused to a promoterless plasmid-encoded *lacZ* gene, as reporters of σ^E^, Cpx, and Rcs regulons, respectively.

*E. coli lacZ^−^* cells, transformed with the reporter plasmids, were treated with thanatin using the same concentration applied in the transcriptomic experiments (corresponding to 1.5 × MIC). Samples were collected 5, 30 and 120 min after treatment with thanatin and tested for β-galactosidase activity. Samples from untreated cells were utilized for baseline correction. As shown in Figure 4, the Cpx system was strongly and significantly activated at 30 and 120 min but not at 5 min. This divergent expression profile at the early time point likely reflects the rapid detection of changes in mRNA abundance by RNA-seq, whereas *lacZ* promoter fusions exhibit delayed and cumulative reporter activity due to the time required for protein synthesis and maturation. The Rcs system was activated at 30 and strongly at 120 min, whereas the activation of the σ^E^ regulon was significant only in cells following 120 min treatment with thanatin. Overall, the Cpx, Rcs and σ^E^ activation profile assessed in thanatin treated cells by *lacZ* promoter fusions is consistent with the same observed by RNA-seq analysis.

**Figure 4:**
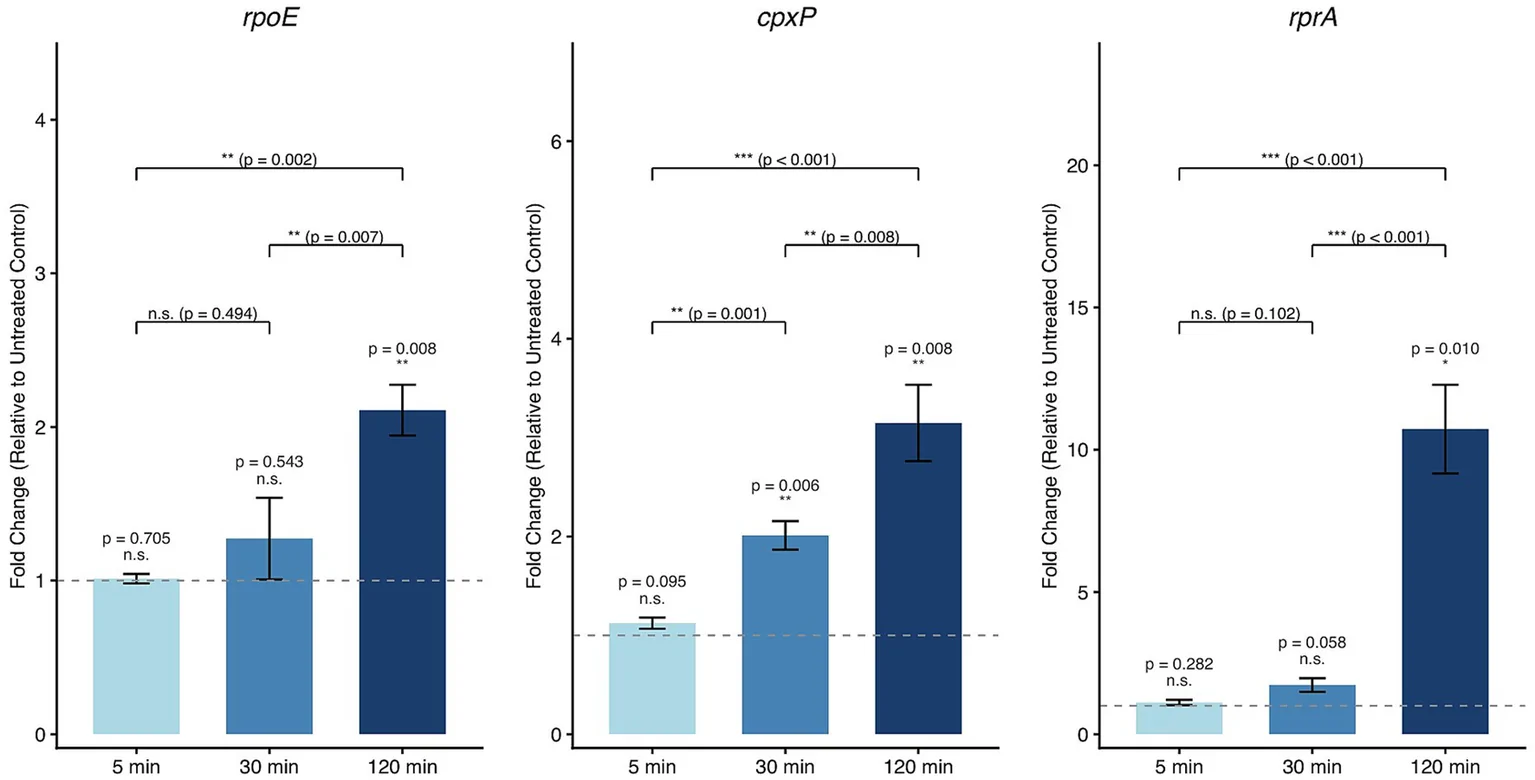
Activation of σE, Cpx and Rcs envelope stress response systems in response to thanatin treatment. β-Galactosidase activity of *rpoE*, *cpxP*, and *rprA* transcriptional reporter fusions in BW25113 cells following thanatin treatment. Activity was measured in Miller units and is expressed as fold change relative to untreated controls. Bars represent mean ± SEM of five independent biological replicates. The dashed line indicates baseline activity (fold change = 1). p-values above each bar indicate two-sided paired *t*-tests against the untreated control (*n* = 5 pairs); brackets indicate pairwise timepoint comparisons. n.s., not significant.

We observed increased expression of *degP*, encoding the periplasmic chaperone/protease DegP, together with decreased expression of *ompF*, which encodes one of the major OM porins. To assess whether these transcriptional changes were reflected at the protein level, we analyzed the steady-state levels of DegP and OmpF in *E. coli* MG1655 cells at 30 and 120 min following thanatin treatment by western blot analysis. In line with transcriptomic data, DegP levels progressively increased upon thanatin exposure, whereas a reduction in OmpF level was observed at 120 min in treated cells compared to untreated controls (Figure 5A).

**Figure 5:**
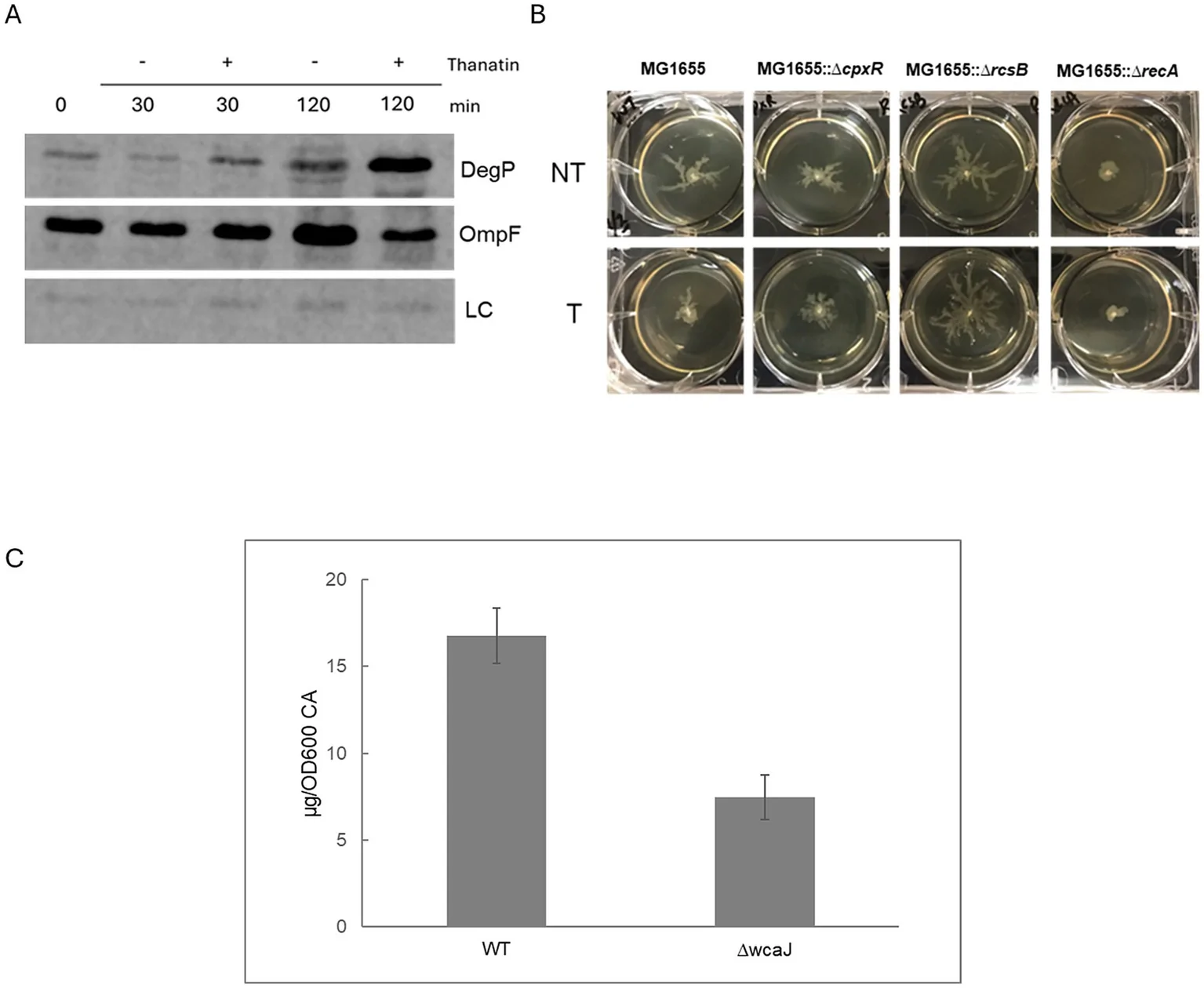
Phenotypic validation of transcriptomics data. **(A)** Steady-state levels of DegP and OmpF in response to thanatin treatment. Samples were collected before (0) and 30 and 120 min after treatment with thanatin. Whole-cell extracts were prepared and analyzed by western blot with anti-DegP and anti-OmpF antibodies. An equal amount of cells (0.36 OD_600_ units) was loaded into each lane. LC is an unknown protein non-specifically recognised by anti OmpF antibody and used as loading control. **(B)** Swarming assay showing motility profile of *E. coli* MG1655 and mutants after 24 hours on swarming agar plates containing (T) or not (NT) thanatin 0.16 × MIC. Representative data from three independent experiments, with technical duplicates are shown. **(C)** Production of colanic acid (CA) normalized on cell mass (OD_600_) in BW25113 wild type strain and Δ*wcaJ* mutant, lacking the enzyme encoding the initial step of CA biosynthesis. No CA was produced by WT and Δ*wcaJ* mutant untreated cells.

Because several genes involved in flagellar biosynthesis were downregulated as early as 30 min after thanatin exposure, we next examined whether this transcriptional response had functional consequences for bacterial motility. To this end, we assessed the effect of thanatin on MG1655 using a swarming assay. Swarming is defined as a coordinated multicellular behavior that enables collective movement across semisolid surfaces and is driven by flagellar rotation (Kearns, 2010). We reasoned that repression of flagellar biosynthesis would result in reduced colony spreading on swarming plates. As shown in Figure 5B, MG1655 cells inoculated onto glucose supplemented swarming plates formed branched, dendritic structures indicative of active swarming motility. In contrast, these structures were absent in colonies of the Δ*recA* strain, which is defective in motility (Gómez-Gómez et al., 2007) and was used as a negative control. We used a sublethal concentration of thanatin to allow bacterial growth while still enabling the observation of thanatin-dependent phenotypes. The presence of thanatin at a concentration corresponding to 0.16xMIC significantly reduced MG1655 colony spreading, consistent with impaired motility. These observations match the transcriptomic downregulation of flagellar genes. The Δ*rcsB* mutant exhibited pronounced hypermotility under both thanatin-treated and untreated conditions. However, the Δ*cpxR* mutant did not display detectable differences in colony spreading under either condition. Together, these results suggest that repression of bacterial motility in response to thanatin is mainly driven by the Rcs stress response and may require coordinated activation of the Cpx system.

Thanatin treatment also induced upregulation of several genes involved in CA biosynthesis. LPS molecules decorated with CA units are known to accumulate upon blockage of LPS transport across the periplasm (Sperandeo et al., 2008). Consistently, increased CA production was confirmed here in BW25113 upon treatment with thanatin by extraction and quantification using a modified Dische colorimetric assay (Dische and Shettles, 1951; Figure 5C). As a control, we employed the Δ*wcaJ* mutant, which lacks the enzyme catalyzing the first committed step of CA biosynthesis. Interestingly, while the untreated strains did not produce detectable levels of CA, treatment with thanatin induced the production of CA in both strains to a higher extent in the wild type than in the Δ*wcaJ* mutant, suggesting that additional overexpressed genes may contribute to exopolysaccharide production under these conditions. CA induction at the same dose was previously reported in MG1655 (Moura et al., 2020).

### 3.6 Effect of thanatin on cell morphology

Thanatin interaction with LptA disrupts the Lpt complex assembly by interfering with LptA-LptC and LptA-LptA interactions, thereby blocking LPS transport (Moura et al., 2020). Impaired LPS transport affects proper cell separation, ultimately leading to the formation of chained cells (Morè et al., 2019). To evaluate the impact of thanatin on cell separation, we examined the morphology of treated cells. Given that our transcriptomic analysis highlighted a central role for the Cpx system in counteracting thanatin-induced damage, we also assessed the morphology in a Δ*cpxR* mutant. Samples from untreated or thanatin-treated cultures were collected after 30 and 120 min and were visualized by confocal laser scanning microscopy following membrane staining with FM5-95 and nucleoid staining with DAPI. In MG1655 wild-type cells, thanatin exposure induced the formation of short chains within 30 min, with the phenotype becoming more pronounced after 120 min. In contrast, thanatin-treated Δ*cpxR* cells did not exhibit a clear chaining phenotype at 30 min, but showed the presence of elongated, non-septated cells after 120 min (Figure 6). To investigate the potential contribution of the Rcs system to this phenotype, we examined the morphology of the isogenic Δ*rcsB* mutant following thanatin treatment. Similar to the wild-type strain, short chains were observed in a fraction of Δ*rcsB* cell population after 30 and 120 min of thanatin treatment (Supplementary Figure S5). Chaining was thus present in wild-type and Δ*rcsB* cells but absent in Δ*cpxR* cells (Figure 6; Supplementary Figure S5).

**Figure 6:**
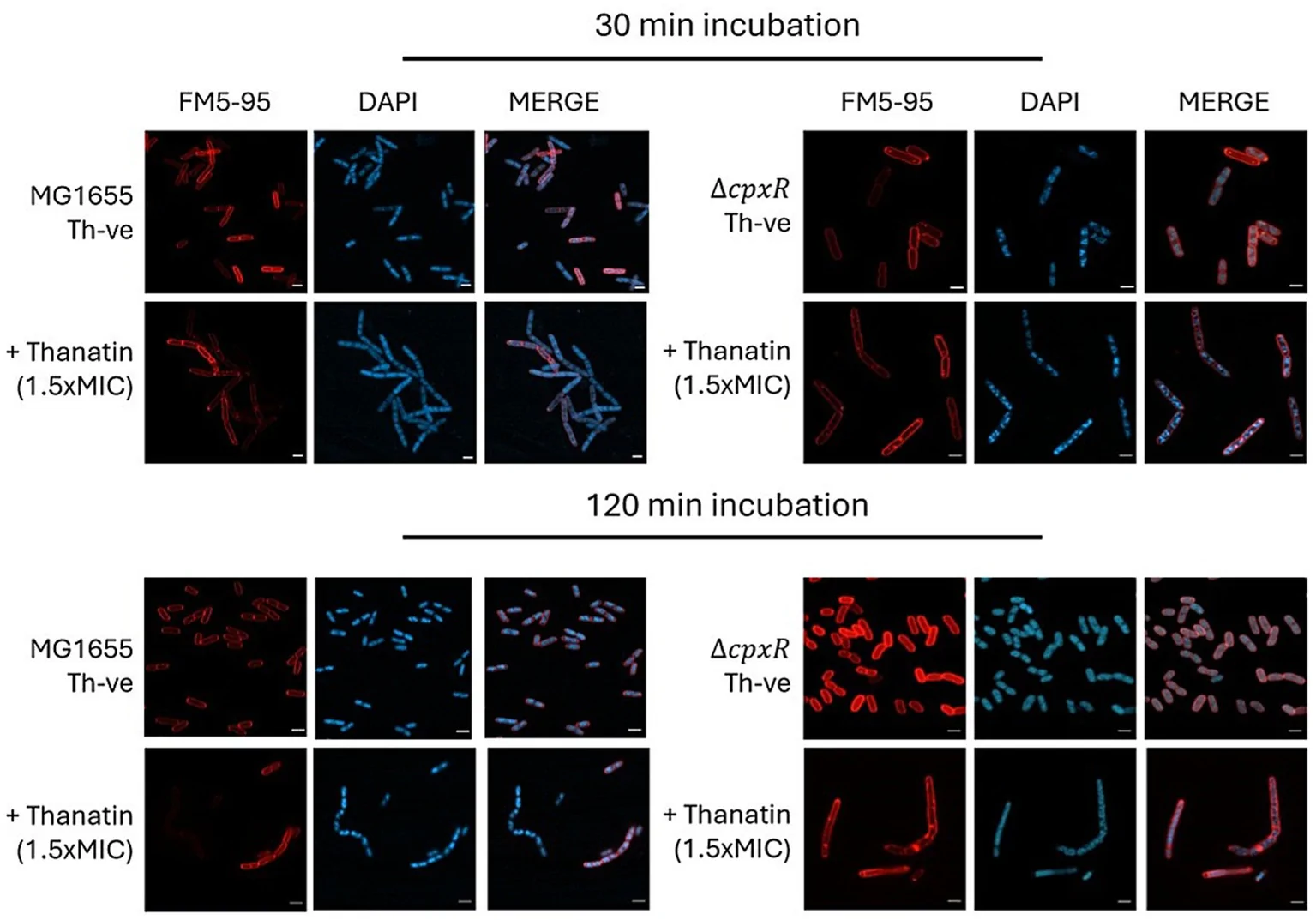
Cell morphology of thanatin treated cells. Confocal microscopy experiments on *E. coli* (MG1655 and Δ*cpxR*) following thanatin exposure. FM5-95 and DAPI staining of MG1655 (left panel) and Δ*cpxR* (right panel) cells after 30 min (Top) or 120 min (Bottom) of exposure to 1.5 × MIC thanatin. Experiments were performed twice in duplicate. Th-ve, thanatin-negative (untreated, water). Scale bar: 2 μm.

## 4 Discussion

This study provides new insights into the global transcriptomic response of *E. coli* MG1655 upon exposure to thanatin, a cationic AMP that directly interacts with LPS (Sinha et al., 2017) at the OM, enters the periplasm and disrupts the assembly of the Lpt machinery, required for LPS transport to the cell surface (Moura et al., 2020; Vetterli et al., 2018). Our results delineate the adaptive mechanisms employed by bacterial cells to counteract thanatin-induced stress: protein quality control and efflux-mediated resistance, reinforcement of the cell wall, and a shift from motility to biofilm formation. They further show that this reinforcement proceeds without any transcriptional induction of the *lpt* genes, identify a Cpx-dependent cell separation defect and an unexpected suppression of the σ^E^ regulon, and describe a late remodelling of OM lipid homeostasis.

Notably, exposure to thanatin modulates the CpxRA and RcsAB ESRs, together with the FlhDC motility regulon. Among these, the CpxRA two-component system represents the earliest activated response, inducing the expression of multiple genes within minutes of treatment. These genes are primarily involved in periplasmic stress adaptation, protein quality control, and efflux pump expression. This early response is likely triggered by the electrostatic interaction between negatively charged LPS molecules and positively charged thanatin molecules and appears to function in restoring osmotic balance via membrane transporters, while reinforcing the cell envelope through increased expression of LdtC, which mediates PG-OM covalent linkage (Magnet et al., 2007). Prolonged exposure to thanatin, corresponding to effective inhibition of LPS transport, results in a stronger induction of the Cpx response. This leads to overexpression of periplasmic chaperones such as Spy and DegP, which mitigate protein misfolding and improper insertion into the IM, alongside a reduction in OmpF expression. This response likely limits the accumulation of unfolded OMPs that cannot be correctly assembled in a compromised OM, while simultaneously reducing porin-mediated influx of toxic compounds. The same Cpx-dependent response also suppresses the periplasmic chaperone Skp via *cpxQ*, a small RNA processed from the *cpxP* 3′ UTR (Mitchell and Silhavy, 2019); since Skp normally escorts unfolded OMPs across the periplasm, its downregulation limits aberrant OMP insertion into the IM (Grabowicz et al., 2016), a second, opposite-direction route to the same protective outcome as Spy/DegP induction. Consistent with this interpretation, the *marRAB* regulon and multiple efflux systems are upregulated. The preservation of IM integrity is further supported by activation of the phage shock protein (Psp) system. Beyond these systems, thanatin also elicits an unexpected suppression of the σ^E^ regulon and a late remodelling of OM lipid homeostasis, considered in turn below.

Beyond the IM, the transcriptional response to LPS depletion reinforces the cell wall rather than attempting to repair the primary lesion: induction of *ldtD* and *ldtC* increases both non-canonical PG crosslinking and covalent Lpp–PG tethering, while the concurrent downregulation of *dpaA* [which normally detaches Lpp from PG (Winkle et al., 2021)] removes the opposing reaction, so the two changes act in the same direction rather than offsetting one another. This coordinated shift builds a more densely crosslinked, more tightly OM-anchored wall, consistent with a defensive strategy that shores up envelope integrity around the existing lesion rather than restoring the transport pathway thanatin itself blocks: none of the seven *lpt* genes changed significantly at any time point.

After 30 min of thanatin exposure, cells activate the Rcs phosphorelay system, which regulates capsule biosynthesis and biofilm formation while repressing motility. Accordingly, genes belonging to the FlhDC regulon are downregulated. This repression is RcsA-mediated (Guttenplan and Kearns, 2013), matches a pattern also reported for other antimicrobials (Rau et al., 2016), and is consistent with the broader repression of flagellar biosynthesis genes observed under diverse envelope- and energy-stress conditions, reflecting the substantial energetic cost of flagellar assembly (Schavemaker and Lynch, 2022). This regulatory assignment is supported genetically: a Δ*rcsB* mutant was hypermotile with and without thanatin, whereas loss of *cpxR* had no effect on colony spreading. This transition from a motile to a sessile lifestyle represents a common bacterial survival strategy under antimicrobial stress, as biofilm formation provides a protective niche that enhances resistance to environmental challenges (Liu et al., 2024).

The capsule itself illustrates this protective role: the negatively charged CA may act as a molecular decoy for thanatin, thereby limiting its interaction with LPS (Llobet et al., 2008), a strategy previously implicated in protection against complement-mediated killing (Miajlovic et al., 2014). As would be expected if demand for exopolysaccharide precursors had risen, the *mglABC* and *gltIJKL* uptake operons were induced over the same interval. CA induction was observed here in BW25113 and had previously been reported in MG1655 at the same dose (Moura et al., 2020), indicating that the response is not restricted to one strain background; induction of *rcsA*, *wzc* and *wcaA* provides the transcriptional basis for this phenotype.

Thanatin treatment induced cell-separation defects. Wild-type cells formed short chains within 30 min, reproducing the phenotype of Lpt component depletion (Morè et al., 2019) and indicating that pharmacological and genetic blockade of LPS transport converge on the same morphological defect. Loss of *cpxR* almost completely abolished this phenotype. Furthermore, in some cases, chaining was replaced by the formation of elongated, non-septated cells, whereas chaining persisted in a Δ*rcsB* background. The cell separation defect is therefore Cpx-dependent rather than Rcs-dependent, matching the early and sustained Cpx activation described above.

Unexpectedly, transcription of the σ^E^ regulon is broadly suppressed or non-induced at 30 min, even though a defect in LPS transport is itself a σ^E^-activating signal. The most economical explanation is the hierarchical relationship between the two responses: CpxR represses the *rpoE-rseA-rseB* operon, so the Cpx activation described above would be expected to limit σ^E^ output even under a stress that would otherwise induce it (Grabowicz and Silhavy, 2017).

Lipid homeostasis at the OM was remodelled over the later part of the time course: induction of the *fad* regulon at 30 and 120 min is consistent with accumulation of phospholipid at the outer leaflet of the OM and is likely triggered by PldA-mediated hydrolysis and subsequent FadR derepression (May and Silhavy, 2018). By contrast, *lplT* and *aas*, the components of the lysophospholipid recycling system that operate downstream of the same PldA-mediated hydrolysis, were not induced, suggesting that β-oxidation rather than lysophospholipid recycling is the dominant route by which cells handle the accumulated phospholipid. The *mla* operon was induced over the same interval (Malinverni and Silhavy, 2009; Low et al., 2021), indicating engagement of retrograde phospholipid transport to restore OM asymmetry. Together, the *fad*/*mla* signature may constitute a metabolic fingerprint indicative of OM perturbation under thanatin treatment.

Thanatin’s transcriptional response can be compared with that of a related antimicrobial peptide: in *E. coli*, resistance to PMB is mediated by the induction of LPS modification enzymes whose expression is controlled by PhoPQ or BaeSR regulatory systems (Trimble et al., 2016). Thanatin, by contrast, did not elicit this route: *eptB*, encoding the phosphoethanolamine transferase that reduces LPS net charge and lowers PMB susceptibility (Acuña et al., 2016), was not induced at any time point. Like PMB (Audrain et al., 2013), we observed that thanatin activates the Rcs and Cpx ESRs, consistent with both peptides perturbing OM integrity. Some components of the response are not thanatin-specific: *osmB* induction is shared with polymyxin B and with a small-molecule inhibitor of LolCDE, the lipoprotein transport machinery that delivers lipoproteins to the OM (Lorenz et al., 2016), a mechanistically unrelated route to envelope stress that strengthens the case for the osmotic arm being a generic consequence of envelope damage rather than an LPS-specific one (Cohen, 2014). However, the broader transcriptional signatures differ: PhoPQ and BaeSR were not detected as differentially expressed under thanatin in this dataset (Supplementary Table S1). Together with each peptide having distinct molecular targets—LptA for thanatin versus LPS headgroup binding for PMB—this indicates overlapping but non-identical adaptive responses for these two LPS targeting compounds. It is worth noting that, unlike thanatin, PMB treatment does not appear to impair Lpt complex assembly, as no degradation of LptA was observed in treated cells (Martorana et al., 2021). These distinct cellular effects may contribute to the different transcriptional responses elicited by the two AMPs.

To our knowledge, this is the first study to characterize the transcriptomic response of *E. coli* to thanatin. Moreover, unlike most transcriptomic analyses of antibiotic-treated bacteria, which are generally limited to a single time point after approximately one generation of growth, our study follows the transcriptional response over the course of treatment. This temporal resolution expands our understanding of thanatin activity by revealing the sequential activation of distinct cellular processes, from early envelope stress responses to later adaptive pathways associated with impaired LPS transport.

Despite the insights provided by this study, several limitations should be acknowledged. First, the transcriptomic analysis was performed under a single growth condition and thanatin concentration, and the observed responses may therefore be influenced by the specific experimental conditions. Second, although our phenotypic analyses support several of the transcriptional changes identified, the precise functional contribution of many differentially expressed genes remains to be established.

## 5 Conclusion

In summary, this study provides novel insights into the bacterial adaptive response to thanatin-induced stress. The observed multifaceted response involves interconnected regulatory networks that coordinate distinct yet complementary mechanisms to preserve envelope integrity and promote survival. Beyond defining the global transcriptional response to thanatin, our study delineates its temporal progression, identifies late adaptive responses involving the *fad* and *mla* pathways, and supports these findings through complementary orthogonal phenotypic analyses. A deeper understanding of these adaptive mechanisms and cellular pathways may inform the development of combination therapies that target both LPS transport and bacterial stress response systems, thereby potentially reducing the emergence of resistance. Future studies should focus on elucidating the functional roles of currently uncharacterized genes and on exploring synergistic interactions between thanatin and other antimicrobials to enhance their therapeutic efficacy.

## Data Availability

The datasets presented in this study can be found in the Gene Expression Omnibus (GEO) repository, accession number GSE271942 (https://www.ncbi.nlm.nih.gov/geo/query/acc.cgi?acc=GSE271942).

## Footnotes

1 http://www.bioinformatics.babraham.ac.uk/projects/fastqc/.
