# Supplementary material for: Time-resolved transcriptomic analysis reveals envelope stress–mediated adaptation of Escherichia coli to the LPS transport inhibitor thanatin
Source: Front Microbiol. 2026 Sep 30;17:1920608. doi: 10.3389/fmicb.2026.1920608 (PMC13661569; doi:10.3389/fmicb.2026.1920608)
Supplement: Supplementary file 1 [file Data_Sheet_1.PDF]

Supplementary Figures

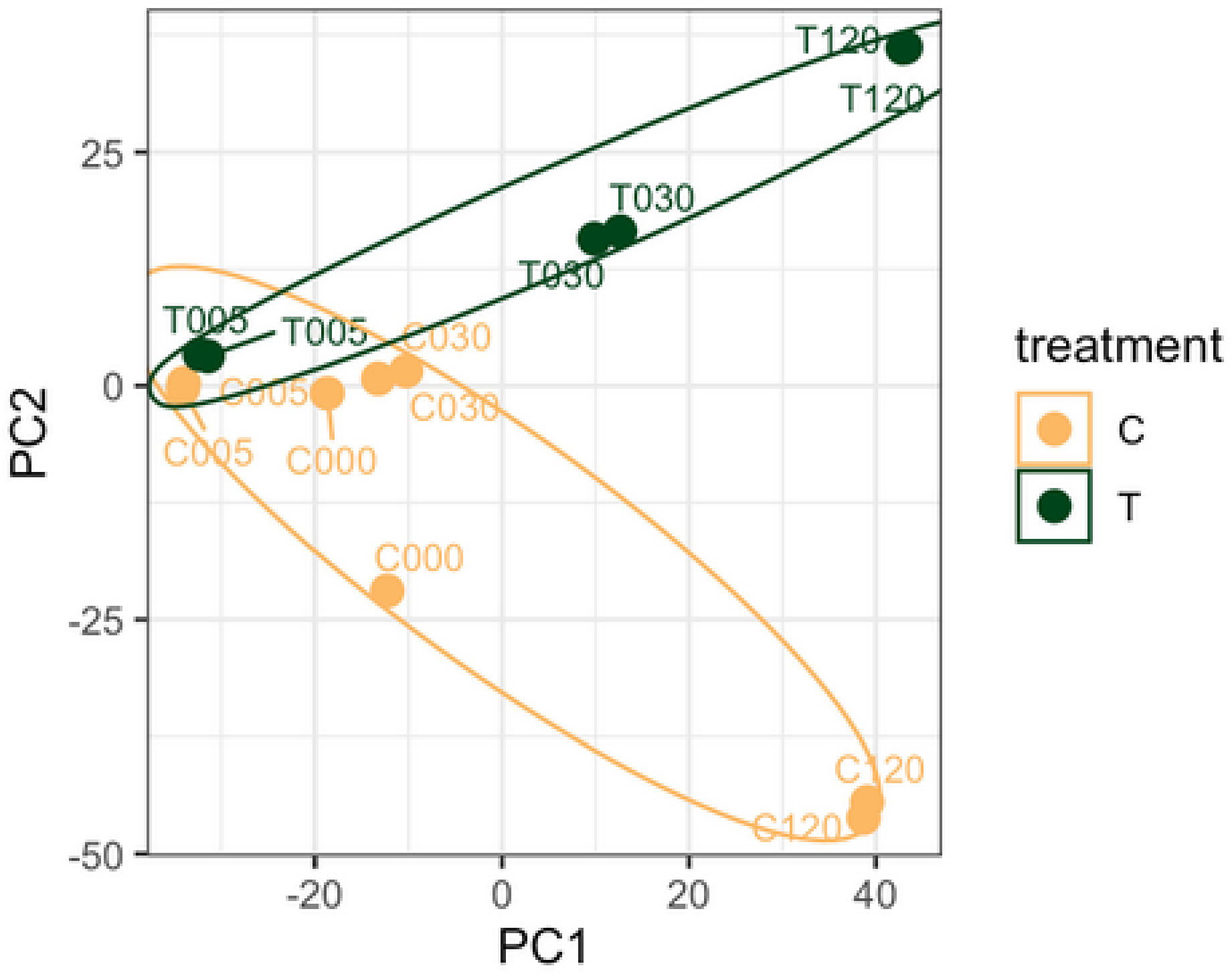

Supplementary Figure S1. RNA-sequencing (Seq) data processing. Principal component analysis (PCA) of the log_2-transformed and normalized count data between different control (C) and treatment (T) samples.

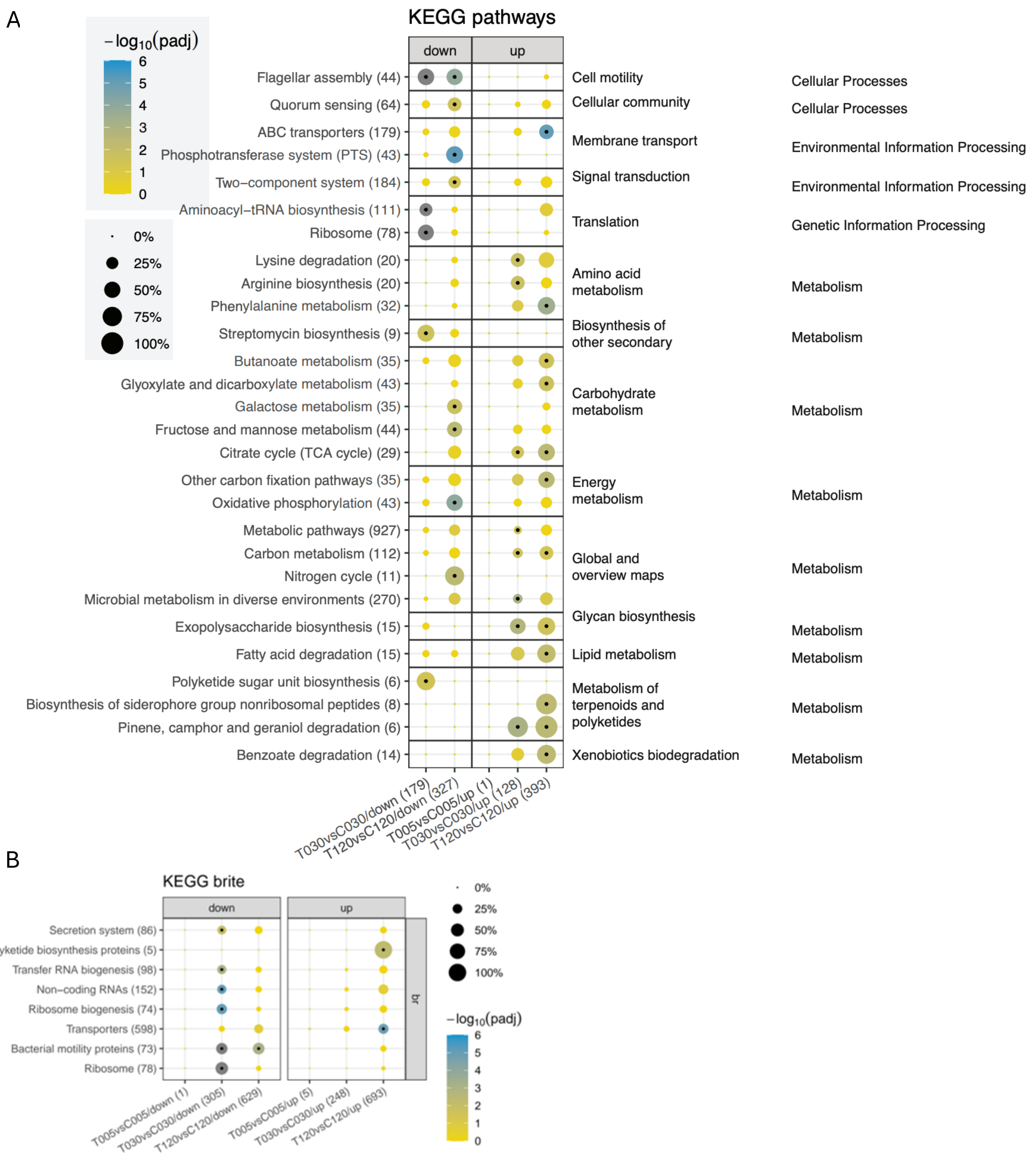

Supplementary Figure S2. Pathway enrichment analysis of significant differentially regulated genes (DEGs) across the three time points under thanatin treatment. (A) Directed KEGG pathway analysis of significant DEGs upon thanatin exposure. **(B)** KEGG brite analysis.

The circle size represents the fraction of the number of DEGs regulated by the corresponding regulon out of the total number of DEGs mapping to the concomitant regulon. The color of the circle represents the significance based on the $Log_{10}P_{adj} < 0.05$. More blue shade represents higher significance and more yellow represents low significance. The black dot in the middle of the circle means $Log_{10}P_{adj} < 0.05$.

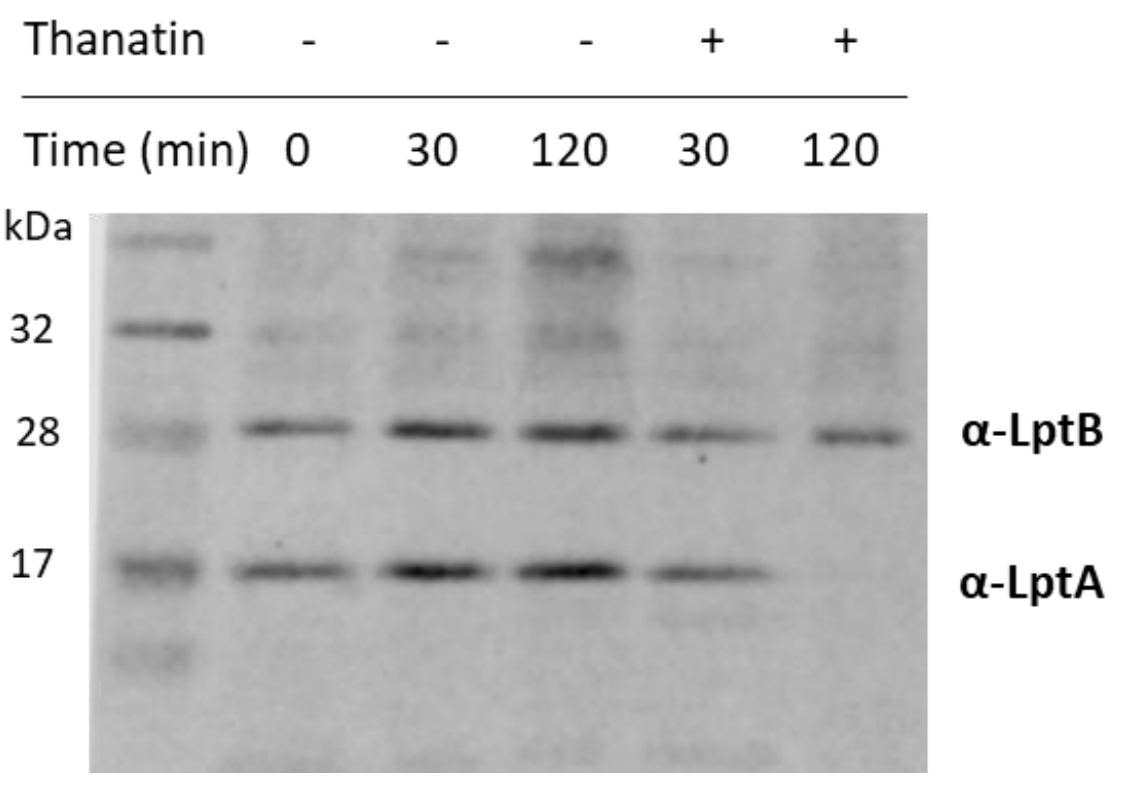

Supplementary Figure S3. Steady-state level of LptA. Cells were collected from the same samples used for RNA extraction at 0, 30 and 120 min after treatment with thanatin to monitor LptA degradation. Whole-cell extracts were prepared and analyzed by western blot with anti-LptA antibodies. An equal amount of cells (0.83 OD_{600} units) was loaded into each lane. As control of specific LptA degradation, the same samples were analyzed by western blot with anti-LptB antibodies.

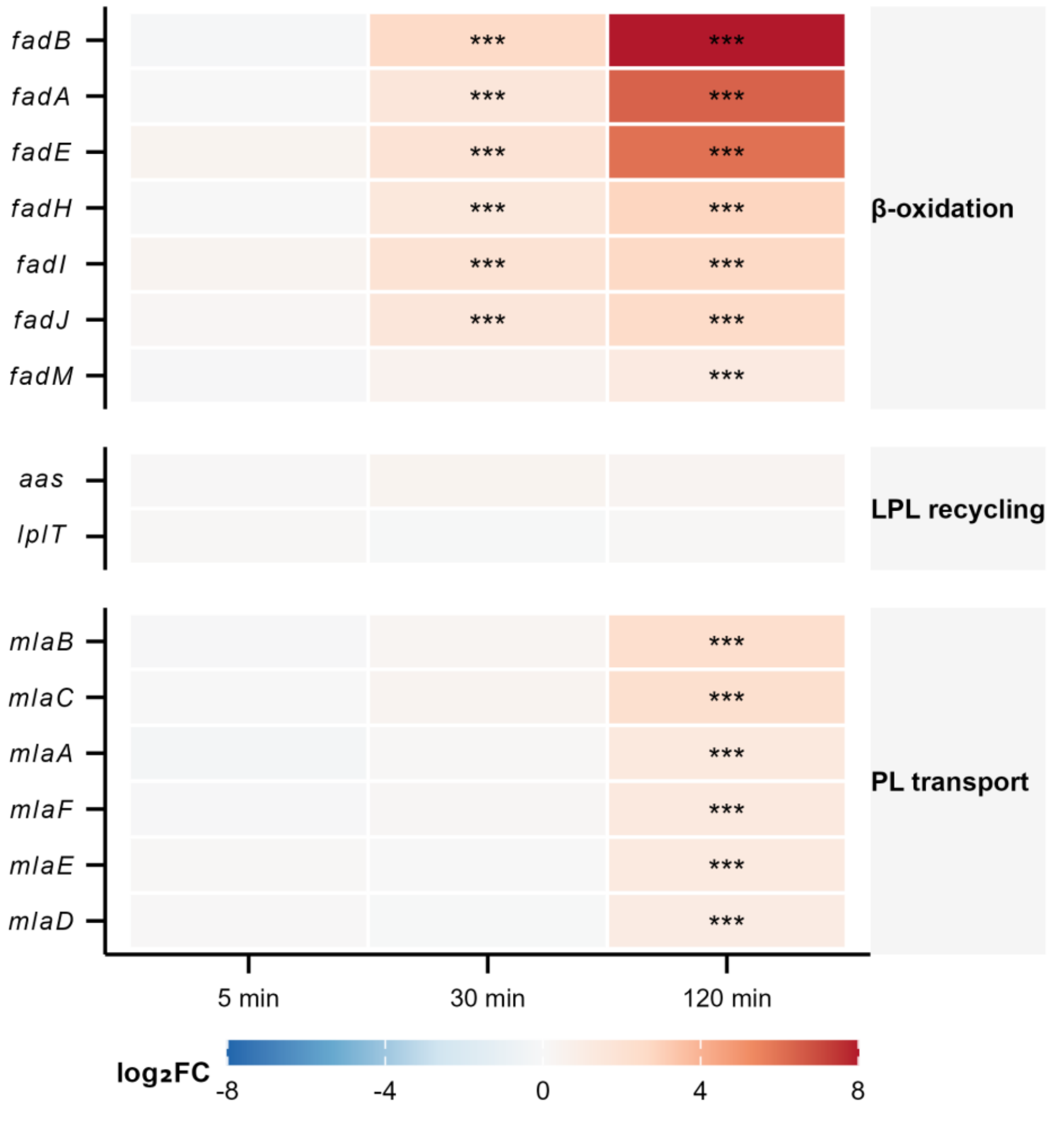

Supplementary Figure S4. Heatmap showing $\log_2$ fold change (thanatin *vs* control) for genes of the *fad* and *mla* operons and for lysophospholipid recycling, at 5, 30, and 120 min of thanatin treatment. Genes are grouped by functional category: β-oxidation (*fadA*, *fadB*, *fadE*, *fadH*, *fadI*, *fadJ*, *fadM*), LPL recycling (*lplT*, *aas*), and phospholipid (PL) transport (*mlaA–mlaF*). Within each group, genes are ordered by $\log_2$FC at 120 min (most induced at top). Colour indicates $\log_2$FC relative to the contemporaneous untreated control; red denotes increased expression and blue denotes decreased expression. Note that key genes implicated in the lysophospholipid recycling system are not differentially expressed at any time point. Significance markers indicate genes meeting both a fold-change threshold ($|\log_2 FC| \geq 1$) and an adjusted p-value threshold: * $p < 0.05$; ** $p < 0.01$; *** $p < 0.001$.

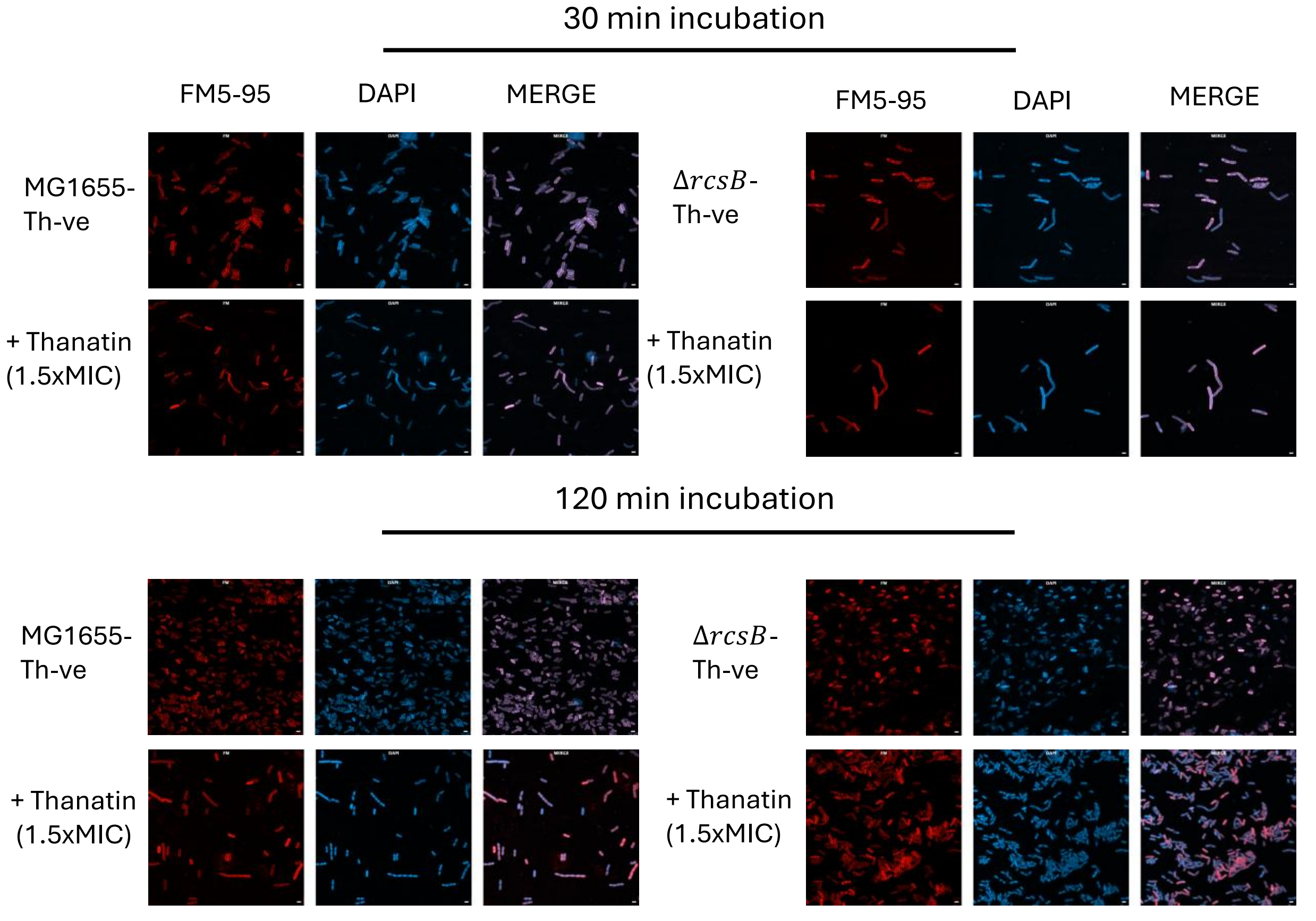

Supplementary Figure S5. Cell morphology of wild-type and *rcsB* mutant thanatin treated cells. Confocal microscopy experiments on *E. coli* (MG1655 and Δ*rcsB*) following thanatin exposure. FM5-95 and DAPI staining of MG1655 (left panel) and Δ*rcsB* (right panel) cells after 30 min (Top) or 120 min (Bottom) of exposure to 1.5xMIC thanatin. Th-ve, water. Scale bar: 2 µm.
